# Cryptochromes modulate E2F family transcription factors

**DOI:** 10.1038/s41598-020-61087-y

**Published:** 2020-03-05

**Authors:** Alanna B. Chan, Anne-Laure Huber, Katja A. Lamia

**Affiliations:** 1 Department of Molecular Medicine, The Scripps Research Institute, 10550 North Torrey Pines Road, La Jolla, CA 92037 USA; 2 Centre de Recherche en Cancerologie de Lyon, 28 rue Laennec, 69008 Lyon, France

**Keywords:** Circadian rhythms, Ubiquitin ligases

## Abstract

Early 2 factor (E2F) family transcription factors participate in myriad cell biological processes including: the cell cycle, DNA repair, apoptosis, development, differentiation, and metabolism. Circadian rhythms influence many of these phenomena. Here we find that a mammalian circadian rhythm component, Cryptochrome 2 (CRY2), regulates E2F family members. Furthermore, CRY1 and CRY2 cooperate with the E3 ligase complex SKP-CULLIN-FBXL3 (SCF^FBXL3^) to reduce E2F steady state protein levels. These findings reveal an unrecognized molecular connection between circadian clocks and cell cycle regulation and highlight another mechanism to maintain appropriate E2F protein levels for proper cell growth.

## Introduction

## Circadian rhythms and the cell cycle

Circadian rhythms allow organisms to anticipate daily environmental changes in accordance with the Earth’s rotation. In mammals, nearly every cell has a circadian clock^1^. Mammalian circadian rhythms are generated by a core molecular clock composed of a transcription translation feedback loop (TTFL); the heterodimer circadian locomotor output cycles kaput (CLOCK) and Brain and muscle ARNT-like protein 1 (BMAL1) drives transcription of their own repressors Period (PER 1–3) and Cryptochrome (CRY1–2). Circadian clocks have been found to influence cell cycle progression, possibly through transcriptional regulation of *c-Myc*, *Ccnd1*, and/or *Wee-1*^2,3^. It is becoming increasingly clear that the circadian clock and the cell cycle can interact dynamically, but the molecular mechanisms connecting these two biological oscillators are largely unknown^2,4,5^.

TTFL components have been postulated to have cellular functions outside circadian rhythm generation^2,6,7^. CRY1/2 bind many thousands of unique sites in the genome independent of CLOCK and BMAL1^8^, and interact with several non-circadian transcription factors including nuclear hormone receptors^9–12^ and c-MYC^13^. In addition, CLOCK, BMAL1, PERs, and CRYs have been implicated in cell cycle control. CLOCK and BMAL1 overexpression in a human colon cancer cell line prevented entry into S phase possibly through decreased CYCLIN D1 protein levels^14^. In wildtype mice subjected to partial hepatectomy (PH), hepatocytes entered M phase faster when the surgery was performed in the afternoon compared to those that underwent PH at night, possibly due to lower *Wee1* expression in the afternoon, suggesting a link between time of day and cell cycle progression^3^. PER has been implicated in cell cycle control via *p16-Ink4A*, which is rhythmically expressed throughout the day and is suppressed by PER2^6^. PER2 also modulates the stability of P53^15,16^, which could influence the cell cycle in unstressed conditions and help the cell anticipate genotoxic stress. Another important protein involved in cell cycle control is c-MYC – a widely known proto-oncogene. We recently discovered that CRY2 acts as a co-factor for the SKP-CULLIN-FBXL3 (SCF^FBXL3^) complex to promote degradation of phosphorylated substrates, including c-MYC^13^ and Tousled-like kinase 2 (TLK2)^17^.

## The E2F family

The Early 2 Factors (E2Fs) are a family of eight winged-helix transcription factors that are key to regulating cell cycle progression from G1 to S phase among other functions^18,19^. E2F1, E2F2, and E2F3a are expressed in early G1 through S phase and are believed to promote S phase initiation by activating genes involved in the G1 to S phase transition^18^. E2F3b, E2F4, E2F6, E2F5, E2F7, and E2F8 act as repressors and are expressed later in the cell cycle^18^. Since E2Fs are crucial in mediating cell cycle progression their protein levels must by tightly regulated. SCF^SKP2^ interacts with the N-terminus of E2F1 and promotes ubiquitination and degradation of E2F1^20^. The anaphase-promoting complex (APC) promotes E2F1 degradation during M phase and this requires interaction between the C-terminus of E2F1 and the APC adaptor protein CDH1^21^. APC/C^CDH1^ also stimulates degradation of E2F3, E2F7, and E2F8^22,23^. Recently, CYCLIN F has been shown to promote ubiquitination of E2F1-3a^24^. Here, we demonstrate that CRY1/2 and SCF^FBXL3^ reduce the steady state protein levels of some E2F family members. These findings highlight a more widespread substrate repertoire of CRY2 and SCF^FBXL3^ mediated degradation and further supports the interconnection between circadian clocks and cell cycle progression.

## Results

### E2F target genes are upregulated in *Cry2*^−/−^ compared to Wildtype MEFs

**Figure 1:**
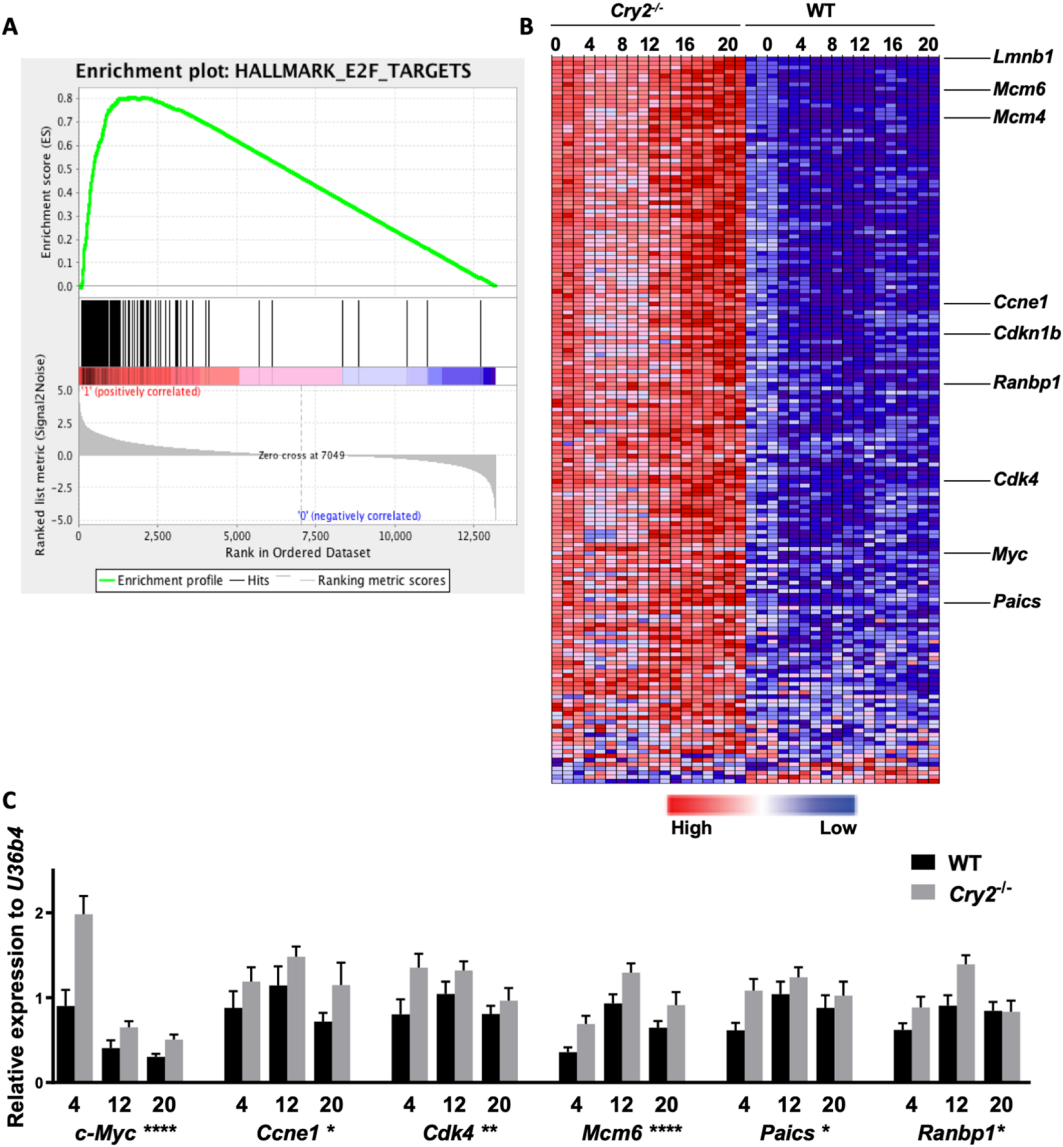
E2F target genes are upregulated in the absence of CRY2. (**A**) GSEA enrichment plot: The top portion shows the running enrichment score (ES) for the gene set as the analysis walks down the ranked list. The middle portion shows where the members of the gene set appear in the ranked list of genes. The bottom portion shows the value of the ranking metric as you move down the list of ranked genes. The ranking metric measures a gene’s correlation with a phenotype (FDR q-value = 0.07). RNA sequencing data from^13^. (**B**) Heatmap from RNA sequencing in primary MEFs at the indicated times after circadian synchronization with dexamethasone. Colors represent high (red) to blue (low) expression. The associated ranked gene names are provided in Table S1. (**C**) Expression of indicated transcripts in primary WT (black) and *Cry2*^−/−^ (gray) MEFs at indicated times (hours) after dexamethasone treatment. Data represent mean ± s.e.m. of 2–3 biological triplicates each analyzed in triplicate. *p < 0.05, **p < 0.01, ****p < 0.0001 by two-way ANOVA with Tukey’s multiple comparisons for effect of genotype.

To determine how the global transcriptome is altered in wildtype (WT) and *Cry2*^−/−^ cells in an unbiased manner, we performed gene set enrichment analysis (GSEA) on RNA-sequencing (RNA-seq) data from WT and *Cry2*^−/−^ mouse embryonic fibroblasts (MEFs). Following synchronization of circadian rhythms with the synthetic glucocorticoid agonist dexamethasone^13^. When analyzed as a group^25^, expression of 200 transcripts defined as hallmark^26^ E2F target genes (Table S1) exhibits a small but highly consistent elevation in *Cry2*^−/−^ MEFs compared to WT (Fig. 1A,B). We used RNA samples isolated from independently synchronized WT and *Cry2*^−/−^ cells to evaluate the reproducibility of this finding by directly measuring the expression of a subset of E2F target genes (Figs. 1C and Fig. S1). The expression of *E2f* family members themselves was largely unchanged in *Cry2*^−/−^ MEFs, though *E2f2* and *E2f7* transcripts tend to be increased late and decreased early in the circadian cycle, respectively (Fig. S1). These data suggest that upregulation of E2F target genes involves a post-transcriptional mechanism.

### E2F family members interact with CRY1, CRY2, and FBXL3

**Figure 2:**
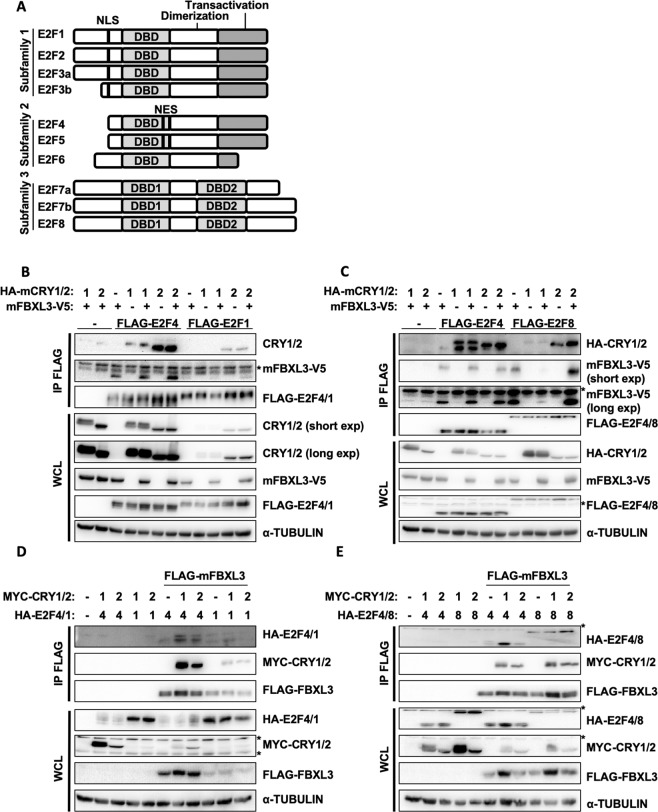
E2F1, E2F4, and E2F8 interact with CRYs and FBXL3. (**A**) Diagram of the three subfamilies of the E2F family, Nuclear localization signal, NLS; DNA binding domain, DBD; Nuclear export signal, NES. (**B**–**E**) Proteins detected by immunoblot (IB) following FLAG immunoprecipitation (IP) or in whole cell lysates (WCL) from HEK293T cells expressing the indicated plasmids with the indicated tags. (*) Denotes non-specific band(s). Short-exp, short exposure; long exp, long exposure. Note: All uncropped blots are provided in Supplementary Information.

As we previously found that CRY2 can act as a co-factor for SCF^FBXL3^ to stimulate ubiquitination and degradation of c-MYC and TLK2^13,17^, we hypothesized that CRY2 could act as a co-factor for E2F family member degradation in a similar manner. We assessed interaction between E2F family members and SCF^FBXL3^ combined with CRY1 or CRY2 using co-immunoprecipitation (co-IP). We investigated E2F1, E2F4, and E2F8 as representatives of the three major E2F subfamilies of activators and repressors (Fig. 2A). Indeed, we found that each of these FLAG-tagged E2F family members, when immunoprecipitated, can interact with FBXL3 and those interactions are enhanced in the presence of CRY1 and CRY2 to varying degrees (Fig. 2B,C). Both E2F1 and E2F8 preferentially interact with CRY2 compared to CRY1, while E2F4 interacts with CRY1 and CRY2. Conversely, when FLAG-tagged FBXL3 is used as bait for immunoprecipitation, its interactions with E2F1, E2F4, or E2F8 are increased in the presence of CRY1 and/or CRY2. E2F4 and E2F8 consistently interact to a greater degree with FBXL3 than does E2F1 (Fig. 2D,E).

### Steady state levels of E2F1, E2F4, and E2F8 are decreased in the presence of CRY2 and FBXL3

**Figure 3:**
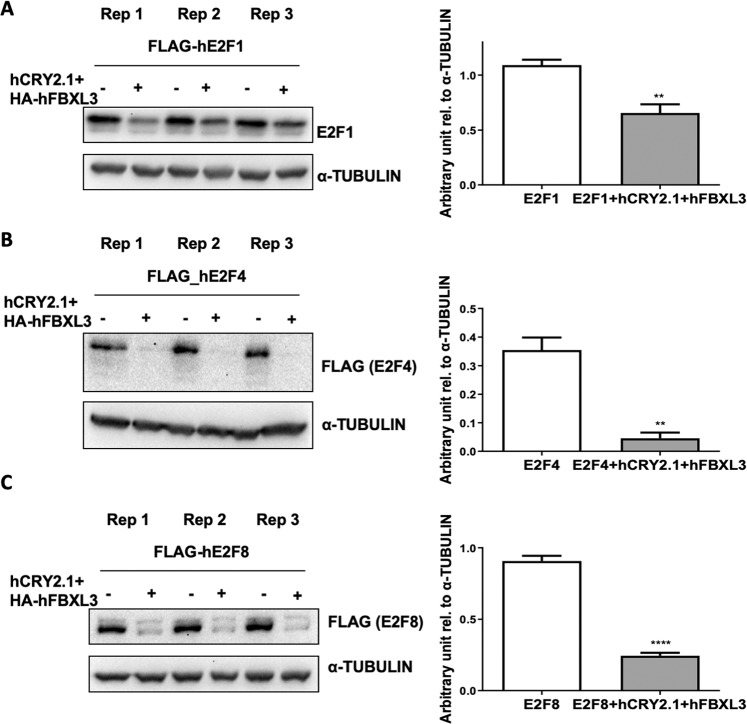
E2F steady state protein levels are affected by CRY2 and FBXL3. (**A**–**C**) Steady state protein levels of FLAG-E2F1, E2F4, and E2F8 in the indicated conditions detected by IB (−/+ hCRY2.1 and HA-hFBXL3). Data in bar graphs indicate mean ± s.e.m. of biological triplicates (Replicate (Rep) 1–3) normalized to α-TUBULIN. **p < 0.01, ****p < 0.0001 using *t*-test.

CRY2 can act as a co-factor to enhance turnover of c-MYC and TLK2 through interaction with FBXL3^13,17^. To determine whether CRY2 and FBXL3 similarly influence E2F family proteins, we measured overexpressed E2F protein levels in the presence or absence of overexpressed human CRY2 (hCRY2.1) and FBXL3. The steady state protein levels of all three E2F proteins studied (E2F1, E2F4, and E2F8) were markedly decreased in the presence of CRY2 and FBXL3 (Fig. 3A–C). This decrease of E2F1, E2F4, and E2F8 protein levels in the presence of human CRY1/CRY2 and FBXL3 was partially mitigated with treatment of MG-132, a reversible proteasome inhibitor. The impact of MG-132 treatment seems to be greatest for E2F4 in the presence of FBXL3 combined with either CRY1 or CRY2 and for E2F8 in the presence of CRY1 and FBXL3 (Fig. S2). The robust effect on steady state protein levels makes it difficult to interpret effects on the turnover of overexpressed proteins, but the turnover of overexpressed E2F1 appears to increase in the presence of overexpressed FBXL3 and CRY2 (Fig. S3).

### Deletion of endogenous *Cry1/2* impacts E2F protein levels

**Figure 4:**
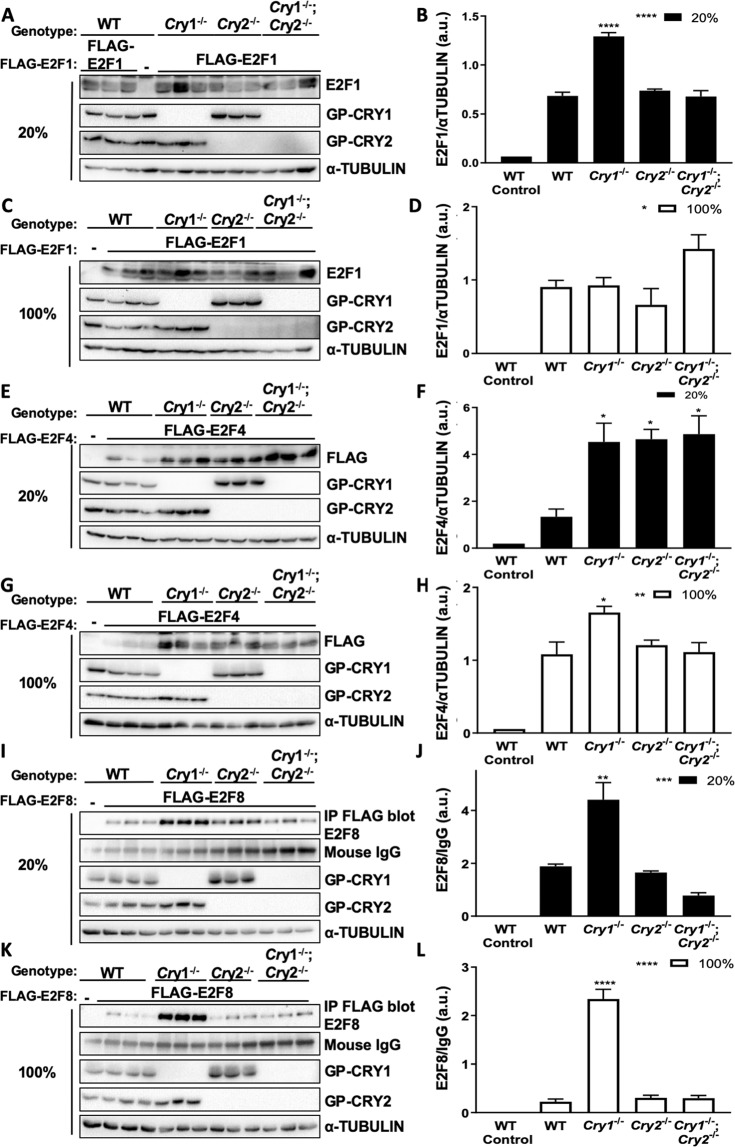
Endogenous CRY1 and CRY2 affect E2F1, E2F4, and E2F8 protein abundance. (**A**,**C**,**E**,**G**,**I**,**K**) Proteins detected by IB in WCL or following FLAG IP (**I**,**K**) from AMEFs of the indicated genotypes stably overexpressing tetracycline-inducible FLAG-tagged human E2F1 (**A**,**C**), E2F4 (**E**,**G**), or E2F8 (**I**,**K**) and treated with 1 µM doxycycline or vehicle (−). For (**I**,**K**), E2F8 protein levels were detected following FLAG IP. (**B**,**D**,**F**,**H**,**J**,**L**) Quantification of data shown in (**A**,**C**,**E**,**G**,**I**,**K**). Data represent the mean ± s.e.m. of three biological replicates for cells plated at 20% (black) or 100% (white) confluency. *p < 0.05, **p < 0.01, ***p < 0.001, ****p < 0.0001 by one-way ANOVA with Dunnett’s multiple comparisons: P values for a main effect of genotype are listed next to legend; P values shown above bars represent post-hoc comparison to WT).

To determine whether endogenous CRY1 and CRY2 impact E2F protein levels, we stably expressed tetracycline-inducible FLAG-tagged human E2F1, E2F4, or E2F8 in WT or *Cry*-deficient adult mouse ear fibroblasts (AMEFs) (Figs. 4 and Fig. S4). Since E2F proteins are involved in cell cycle regulation, we induced their expression at low and high plating densities (20% and 100%) to capture both proliferating and growth-arrested conditions. E2F1 protein levels are elevated when *Cry1* is deleted, but not when *Cry2* or both *Cry1* and Cry2 are deleted at both plating densities (Fig. 4A–D). Even though E2F1 protein abundance is significantly increased in the absence of *Cry1* (Fig. 4B,D), the E2F1 protein levels are highly variable, and loss of Cry1 also seems to impact expression of exogenous *E2F1* mRNA (Fig. S5) making us less confident in the biological significance of the increased E2F1 in the *Cry1*^−/−^ genotype. E2F4 protein is robustly increased in cells lacking either CRY1 or CRY2, regardless of confluency (Fig. 4E–H). E2F8 protein levels are dramatically increased in the absence of *Cry1*, regardless of cell confluency (Fig. 4I–L) suggesting that CRY1 may play a more important role than CRY2 in the regulation of E2F8 protein abundance. We tried to detect endogenous mouse E2F1, E2F4, and E2F8 protein levels in AMEFs; however, we could not confidently detect endogenous mouse E2F1, E2F4, or E2F8. Although we were not able to confirm its specificity with shRNA or E2F8^−/−^ cells, the anti-E2F8 antibody detects a protein at the correct molecular weight (indicated by arrow), which is increased in all *Cry1/2*-deficient genotypes, further supporting our findings regarding E2F8 (Fig. S6). *Cry1/2* genotype had little to no effect on the expression of endogenous *E2f1* or *E2f4* mRNA (Fig. S5). Interestingly, as we have observed for other doxycycline-inducible systems^17^, doxycycline-induced expression of human *E2F1* or *E2F4* mRNA was sometimes elevated in *Cry*-deficient cells compared to WT (Fig. S5). Because the upregulation of doxycycline-induced expression of human *E2F4* mRNA across the genotypes did not follow the same trends observed at the protein level (Fig. 4), CRYs seem to impact E2F4 protein abundance post-translationally. The robust impact on E2F8 protein level suggests that it may also be a target of CRY1/2-dependent post-translational regulation. All in all, these data support our hypothesis that CRY1 or CRY2 can decrease E2F4 and E2F8 protein levels by recruiting them to SCF^FBXL3^.

## Discussion

We find that E2F target genes are slightly but consistently upregulated in *Cry2*^−/−^ MEFs compared to matched WT control cells. *Cry2*^−/−^ MEFs proliferate faster than WT MEFs^13^, which may be effected by higher expression of c-MYC as we have previously documented^13^ and could also be influenced by enhanced E2F activity. CRY2 could also influence cell growth through its partner PER2, which has been found to modulate the stability and nuclear translocation of p53^15,27^, and/or through the actions of the core clock activating complex of CLOCK and BMAL1. It is likely that CRY2 acts in multiple ways to influence cell growth; dissecting the relative contributions of each of these mechanisms to increased proliferation in CRY2-deficient cells will require extensive additional investigation.

Here, we find that both activator and repressor E2F family transcription factors interact with FBXL3 and these interactions are enhanced by CRY1 and/or CRY2. Intriguingly, the representative members of the repressor subfamilies, E2F4 and E2F8, interact more strongly with CRYs and FBXL3 than does the canonical activator E2F1. While we did not identify the region(s) of the E2F proteins that interact with FBXL3 and/or CRYs in this study, these differences likely reflect preferential interaction with amino acid sequences that are conserved in the repressor subfamilies. These observations are also consistent with the greater impact of CRY1/2 overexpression or genetic deletion on protein levels of overexpressed or endogenous repressor E2F family members, E2F4 and E2F8, compared to the activator E2F1.

The observed greater sensitivity of steady state protein levels of repressor E2F family members to the loss of *Cry2* is counterintuitive in the context of the significant elevation of E2F target gene expression in *Cry2*^−/−^ MEFs. However, we only assessed the impact of CRY regulation of a select subset of the E2F family; it is possible that E2F2 and/or E2F3 are significantly stabilized in the absence of CRY2 and contribute to the observed effects on gene expression. Conversely, other E2F repressors may be more responsive to the genetic manipulation of *Cry*s than E2F4 and E2F8 are. Given that CRYs repress the transcriptional activity of CLOCK-BMAL1^28^ and of several nuclear hormone receptors^9^, CRYs may suppress the transcriptional activity of activator E2Fs or directly mediate transcriptional repression conferred by repressor E2Fs. If they do, that could also explain the increased expression of E2F target genes in *Cry2*^−/−^ cells. Indeed, analyzing Chromatin-immunoprecipitation (ChIP) sequencing data from mouse livers across circadian time (GSE39860)^8^ reveals that endogenous CRY1 and CRY2 are associated with the genomic loci containing many of the 200 “hallmark” E2F target genes in mouse livers, and at least some of these sites are not bound by other circadian transcription factors, supporting the idea that CRYs could independently regulate E2F family members bound to chromatin (Table S2). Additionally, E2Fs could have unexpected transcriptional activities in the context of CRY-deficient cells. Finally, some evidence suggests that E2F family members cannot be easily classified into activators and repressors^29^, and the increased expression of E2F target genes in *Cry2*^−/−^ AMEFs may reflect increased target gene activation by E2F family members that are thought to act as repressors.

A recent study demonstrated that CYCLIN F interacts with and promotes the degradation of activator E2Fs to restrict E2F activity to the S phase of the cell cycle^24^. Our finding that CRYs modulate E2F protein abundance suggests a mechanism by which their abundance and activity could be regulated by circadian cycles. Disruption of circadian rhythms, such as that experienced by shift workers, increases the risk of cancer and other pathologies^30^. E2Fs have been implicated in cancer^18,30–32^, and disruption of their modulation by CRYs could contribute to the elevated cancer risk caused by circadian disruption.

## Methods

### Cell culture

Cell culture methods were the same as in^17^. In brief, all primary mouse embryonic (MEF) and adult mouse ear (AMEF) fibroblasts cells were prepared from embryos collected at E15.5 or from ear biopsies of adult mice respectively, and were passaged no more than 10 times as in^17^. Cells were grown in complete Dulbecco’s Modified Eagle Medium (DMEM) (Invitrogen cat #10569) supplemented with 10% fetal bovine serum (FBS) (HEK 293T cells) or 15% FBS (MEFs and AMEFs), and 1% penicillin and streptomycin. HEK 293T cells were grown in a 37 °C incubator maintained at 5% CO_2_ and 20%O_2_ (high oxygen) and MEF cells were grown in a 37 °C incubator maintained at 5% CO_2_ and 3% O_2_ (low oxygen). Transfections were carried out using polyethylenimine (PEI; Polysciences Inc catalog #23966-2) by standard protocols. After 24 hours, the HEK 293T cell media was again replaced and protein extracts were isolated 48 hours post transfection. Cycloheximide (CHX) (Fisher cat# 50255724) was used at a concentration of 100 µg/mL as indicated. Cells were treated with 10 μM MG-132 (Sigma cat# C2211-5MG) for 4–5 hrs prior to lysis.

### Generation of viruses and stable cell lines

Methods were the same as in^17^ except lentiviruses expressing pCW-2xFLAG-E2F1 E2F4, or E2F8 were used.

### Doxycycline induction of stable cell lines

Methods were the same as in^17^: AMEF cells of the indicated genotypes stably expressing pCW-2xFLAG-E2F1, pCW-2xFLAG-E2F4, or pCW-2xFLAG-E2F8 were treated with 1 μM doxycycline (Sigma cat # D9891) for 48 hours to induce 2xFLAG-E2F1 or 2xFLAG-E2F4 expression.

### Immunoprecipitation and western blotting

HEK 293T whole cell extracts for co-IP were prepared using lysis buffer containing 1%TX-100, 50 mM HEPES (pH 7.4), 138 mM KCl, 4 mM NaCl, 50 mM sodium pyrophosphate, 100 mM sodium fluoride, 10 mM EDTA, 1 mM EGTA pH 8, 50 μM PMSF, 1 mM β-Glycerophosphate, 1 mM sodium orthovanadate, and protease inhibitors (Thermo Scientific cat # 88265) as previously described (Lamia *et al*., 2004). Immunoprecipitation was performed using anti-Flag M2 agarose beads (Sigma cat #A2220). Antibodies for Western Blots were anti-FLAG polyclonal (Sigma cat #F7425), anti-V5 polyclonal (Bethyl Labs cat# A190–120A), anti-HA polyclonal (Sigma cat # H6908), anti-α-TUBULIN (Sigma cat # T5168), anti-E2F1 (Santa Cruz KH95), anti-E2F8 (Abcam ab109596), CRY1-CT and CRY2-CT^11^. For CHX assay, HEK293T cells were lysed with a RIPA Buffer containing 1% TX-100, 147 mM NaCl, 12 mM Sodium deoxycholate, 0.1% SDS, 50 mM Tris pH 8.0, 10 mM EDTA, 50 μM PMSF, 1 mM β-Glycerophosphate, 1 mM Sodium Orthovanadate and protease inhibitors (Thermo Scientific cat # 88265).

### Plasmids

pcDNA3-HA-mCRY2, pcDNA3-HA-mCRY1, and pcDNA3-FBXL3-V5 are as previously described^13^. cDNA encoding human *E2F1, E2F4, E2F8, hCRY2.1*, and *FBXL3* were generated by RT-PCR from RNA extracted from HEK 293T cells. *E2F1, E2F4, E2F8, hCRY2.1*, and *FBXL3* cDNAs were cloned into pcDNA3.1-based FLAG-epitope tagged vector or pcDNA3.1-based HA-epitope tagged vector using standard protocols. The FLAG-epitope tag was removed from pcDNA3.1-based FLAG-epitope tagged hCRY2.1 using Q5 Site-Directed Mutagenesis (New England Biolabs Inc. cat # E0554S). psPAX plasmid (Addgene plasmid 12260) and pMD2.G plasmid (Addgene plasmid 12259) deposited by Dr. Didier Trono, and used for infection, were purchased from Addgene. pCW-Cas9 was a gift from Eric Lander & David Sabatini (Addgene plasmid # 50661)^33^ and the *Cas9* sequence was replaced with 2x-FLAG-*E2F1, E2F4, or E2F8* cDNA using the Gibson Assembly Ultra Kit (Synthetic Genomics Inc. cat # GA1200-10).

### Quantitative RT-PCR (qPCR)

Methods were the same as in^17^.

**Primers used for qPCR**

**Table:** 

primer name	Forward (5′-3′)	Reverse (3′-5′)
*m-E2f1*	TGCAGAAACGGCGCATCTAT	CCGCTTACCAATCCCCACC
*h-E2F1*	GAGAACAGGGCCACTGACTCTGCC	GCCGGAGAAGTCCTCCCGCAC
*h-E2F4*	CCCATATGGCGGAGGCCGGGCC	CGCCGCTTCTGGCGTACAGCTAGGG
*m-E2f4*	GGAGCTGCAGCAACGAGAGC	CTAGACTGGTGCCCGATGGC
*m-U36b4*	AGATGCAGCAGATCCGCA	GTTCTTGCCCATCAGCACC
*m-Mcm4*	GAGGAAAGCAGGTCGTCACC	AGGGCTGGAAAACAAGGCATT
*m-Mcm6*	CCTGTGAATAGGTTCAACGGC	CATTTTCCTGAGGTGGAGCAC
*m-Ccne1*	AGCGAGGATAGCAGTCAGCC	GGTGGTCTGATTTTCCGAGG
*m-Cdkn1b*	ACCCGCCCGAGGAGGAAGAT	CTCGCTTCTTCCATATCCCG
*m-Paics*	CAGTTGTTACAGGAAGCTGG	CGTCCTTGAAGAACATCTCC
*m-Lmnb1*	GCTGCTGCTCAATTATGCCAAG	GATGCTTCTAGCTGGGCAATC
*m-Ranbp1*	GATGCGTGCAAAGCTGTTCC	GGTGTAATATAGTGGTTGGCGC
*m-E2f2*	GTGACCTACCAGGATATCCGTG	GCC TTG ACC GCA ATC ACT GTC
*m-E2f3a*	GCCTCTACACCACGCCACAAG	TCGCCCAGTTCCAGCCTTC
*m-E2f3b*	CGGAAATGCCCTTACAGC	CTCAGTCACTTCTTTGGACAG
*m-E2f4*	GGAGCTGCAGCAACGAGAGC	CTAGACTGGTGCCCGATGGC
*m-E2f5*	GTGATGGAAGACTCCATTAATAAC	GGCCCTGAGTGACTCTTC
*m-E2f6*	GGCATCGAACTGGTGGAAAAG	CCAACAGTTGCTGAGCACAATC
*m-E2f7*	GAAGTCTGGCGGCCATCTAC	GACCATTCTGCGCAGAGAAGG
*m-E2f8*	CCCTGTCAAGAGCAACAAAGC	CTG TAG GGT CCA GGG GAG
*m-Myc*	GCGACTCTGAAGAAGAGCAAG	GCCTCGGGATGGAGATGAG
*m-Bmal1*	TCAAGACGACATAGGACACCT	GGACATTGGCTAAAACAACAGTG
*m-Nr1d1 (RevErba)*	CGTTCGCATCAATCGCAACC	GATGTGGAGTAGGTGAGGTC
*m-E2f1*	AGGGAAAGGTGTGAAATCTCC	TTGGTGATGACATAGATGCGC
*m-Cry2* ^−/−^	CCTGGATGCCGATTTCAGTG	ATCGAGAGGGGAAGCCTTTC

### Gene Set Enrichment Analysis (GSEA)

GSEA is a computational method^34^ that determines whether an a priori defined set of genes shows statistically significant, concordant differences between two biological states (e.g., phenotypes). RNA sequencing outputs for WT and *Cry2*^−/−^ cells were used as input for GSEA comparing the two genotypes (WT versus *Cry2*^−/−^ regardless of circadian time).

### Mice

*Cry1*^−/−^; *Cry2*^−/−^ mice from which primary cells were derived were from Dr. Aziz Sancar^43^. All animal care and treatments were in accordance with Scripps Research guidelines and regulations for the care and use of animals. All procedures involving experimental animals were approved by the Scripps Research Institutional Animal Care and Use Committee (IACUC) under protocol #10-0019.

### Accession numbers

The accession number for the RNA sequencing data analyzed in this paper is GEO: GSE89018.

### Statistical analysis

Statistics for Fig. 1A were calculated in the Gene Set Enrichment Analysis program as described^34^. Graph Pad Prism 7 was used to compute all other statistics. For Figs. 1C, S1–3 two-way ANOVA with Tukey’s multiple comparisons was used. For Fig. 4B,D,F,H,J,L one-way ANOVA with Dunnett’s multiple comparisons was used. For Fig. 3, *t*-test was used.

## Supplementary information

Supplementary information.

Supplementary information2.
