# Supplementary material for: Cryptochromes modulate E2F family transcription factors
Source: Sci Rep. 2020 Mar 5;10:4077. doi: 10.1038/s41598-020-61087-y (PMC7058038; doi:10.1038/s41598-020-61087-y)
Supplement: Supplementary file 1 — Supplementary information. [file 41598_2020_61087_MOESM1_ESM.pptx]

## Slide 1
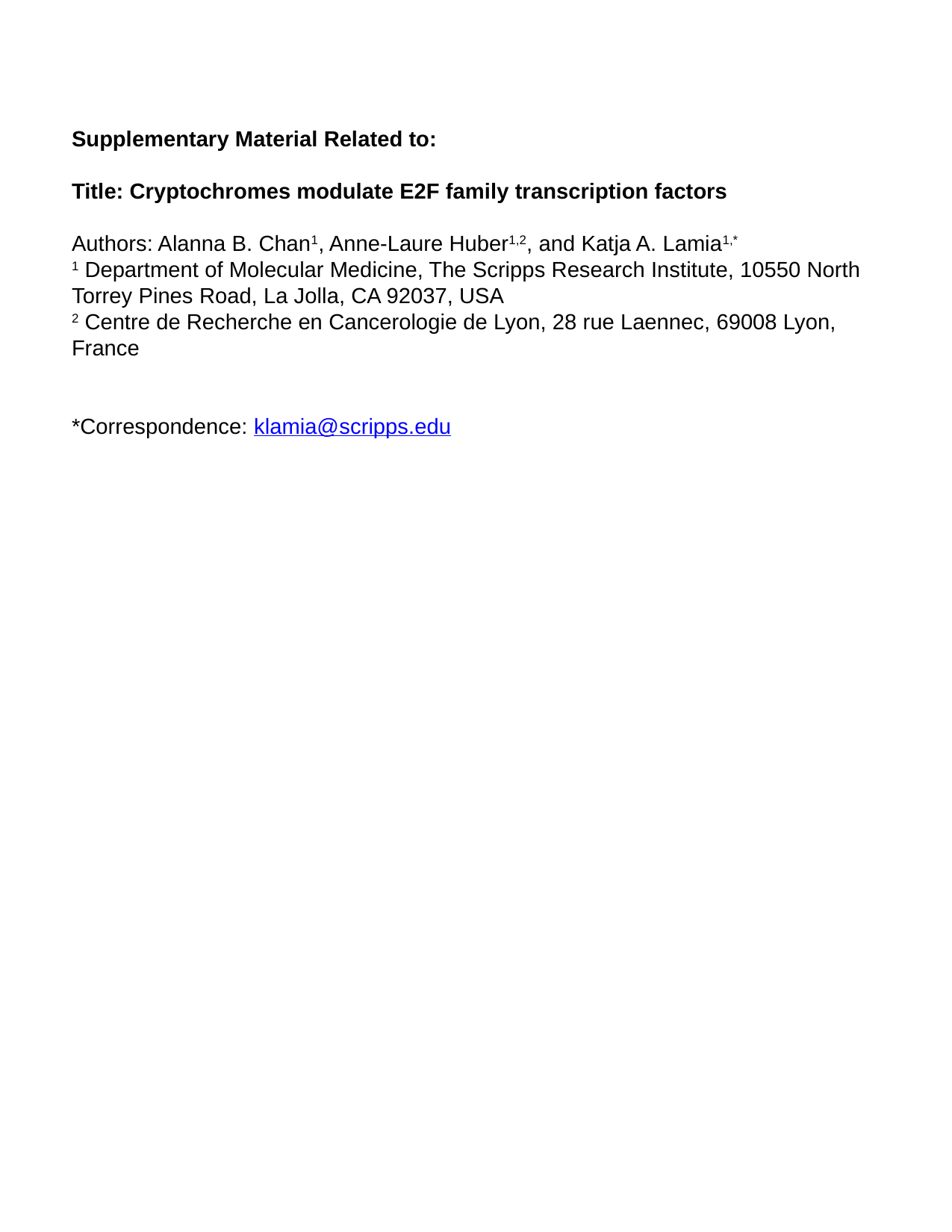

Supplementary Material Related to:
Title: Cryptochromes modulate E2F family transcription factors
Authors: Alanna B. Chan1, Anne-Laure Huber1,2, and Katja A. Lamia1,*
1 Department of Molecular Medicine, The Scripps Research Institute, 10550 North Torrey Pines Road, La Jolla, CA 92037, USA
2 Centre de Recherche en Cancerologie de Lyon, 28 rue Laennec, 69008 Lyon, France
*Correspondence: klamia@scripps.edu

## Slide 2
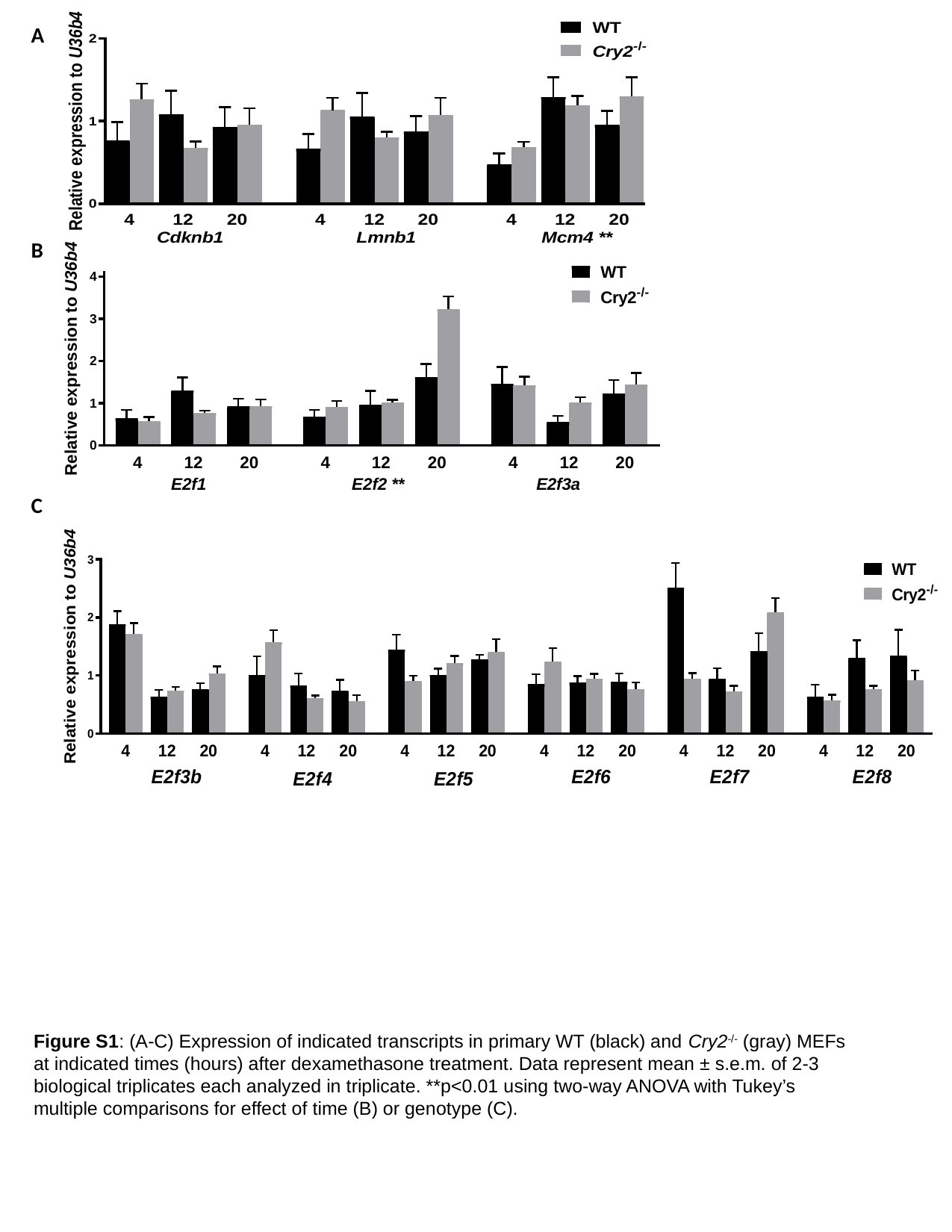

A
B
C
Figure S1: (A-C) Expression of indicated transcripts in primary WT (black) and Cry2-/- (gray) MEFs at indicated times (hours) after dexamethasone treatment. Data represent mean ± s.e.m. of 2-3 biological triplicates each analyzed in triplicate. **p<0.01 using two-way ANOVA with Tukey’s multiple comparisons for effect of time (B) or genotype (C).

## Slide 3
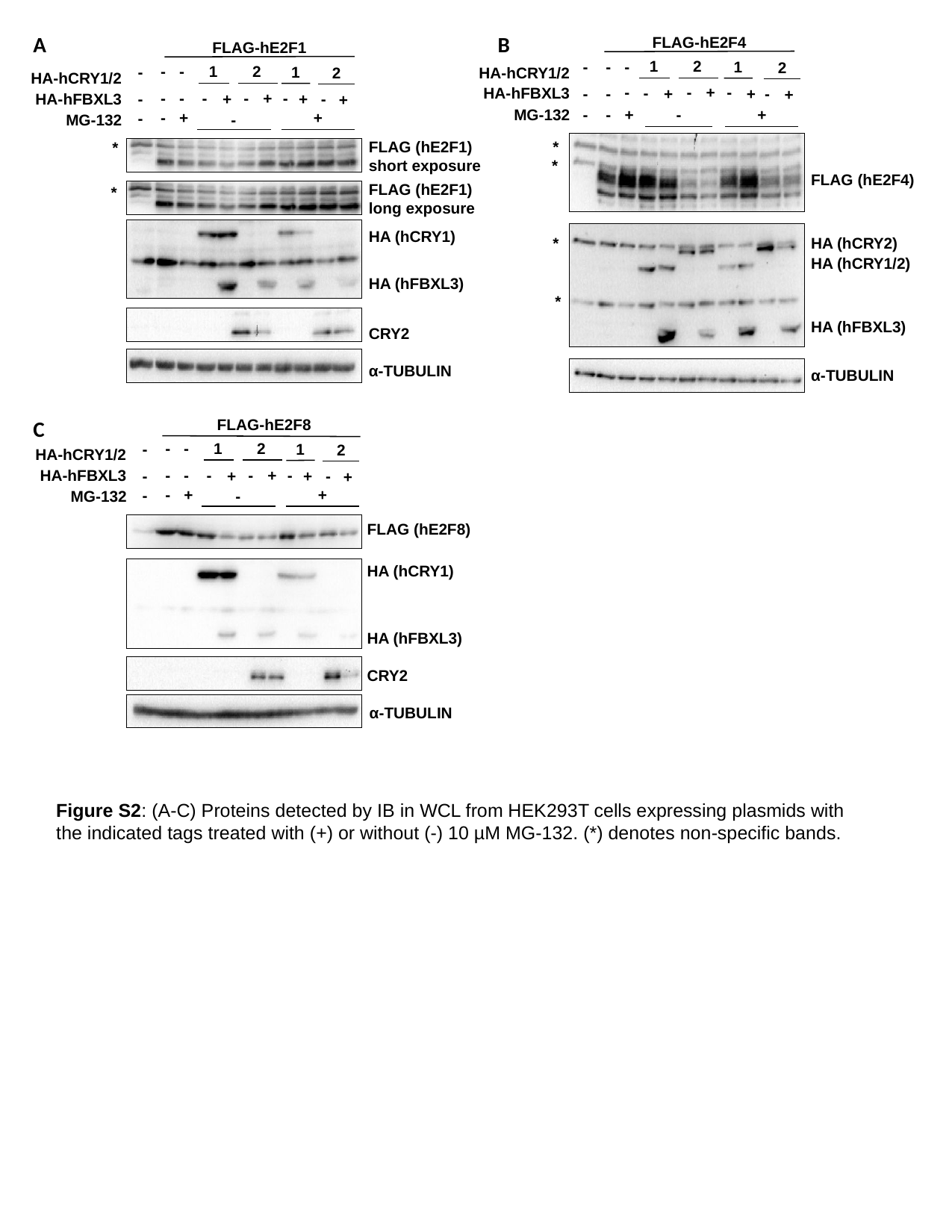

A
B
FLAG-hE2F4
FLAG-hE2F1
2
1
-
1
-
-
2
2
-
-
1
-
1
HA-hCRY1/2
2
HA-hCRY1/2
-
-
+
-
-
HA-hFBXL3
+
-
-
-
+
+
-
-
-
-
-
+
HA-hFBXL3
+
-
-
+
+
MG-132
-
-
+
-
+
-
+
+
-
-
MG-132
FLAG (hE2F1) short exposure
*
*
*
FLAG (hE2F4)
FLAG (hE2F1)
long exposure
*
HA (hCRY1)
HA (hCRY2)
*
HA (hCRY1/2)
HA (hFBXL3)
*
HA (hFBXL3)
CRY2
α-TUBULIN
α-TUBULIN
C
FLAG-hE2F8
2
-
-
1
-
1
2
HA-hCRY1/2
-
-
-
-
-
+
HA-hFBXL3
+
-
-
+
+
-
+
+
-
-
MG-132
FLAG (hE2F8)
HA (hCRY1)
HA (hFBXL3)
CRY2
α-TUBULIN
Figure S2: (A-C) Proteins detected by IB in WCL from HEK293T cells expressing plasmids with the indicated tags treated with (+) or without (-) 10 µM MG-132. (*) denotes non-specific bands.

## Slide 4
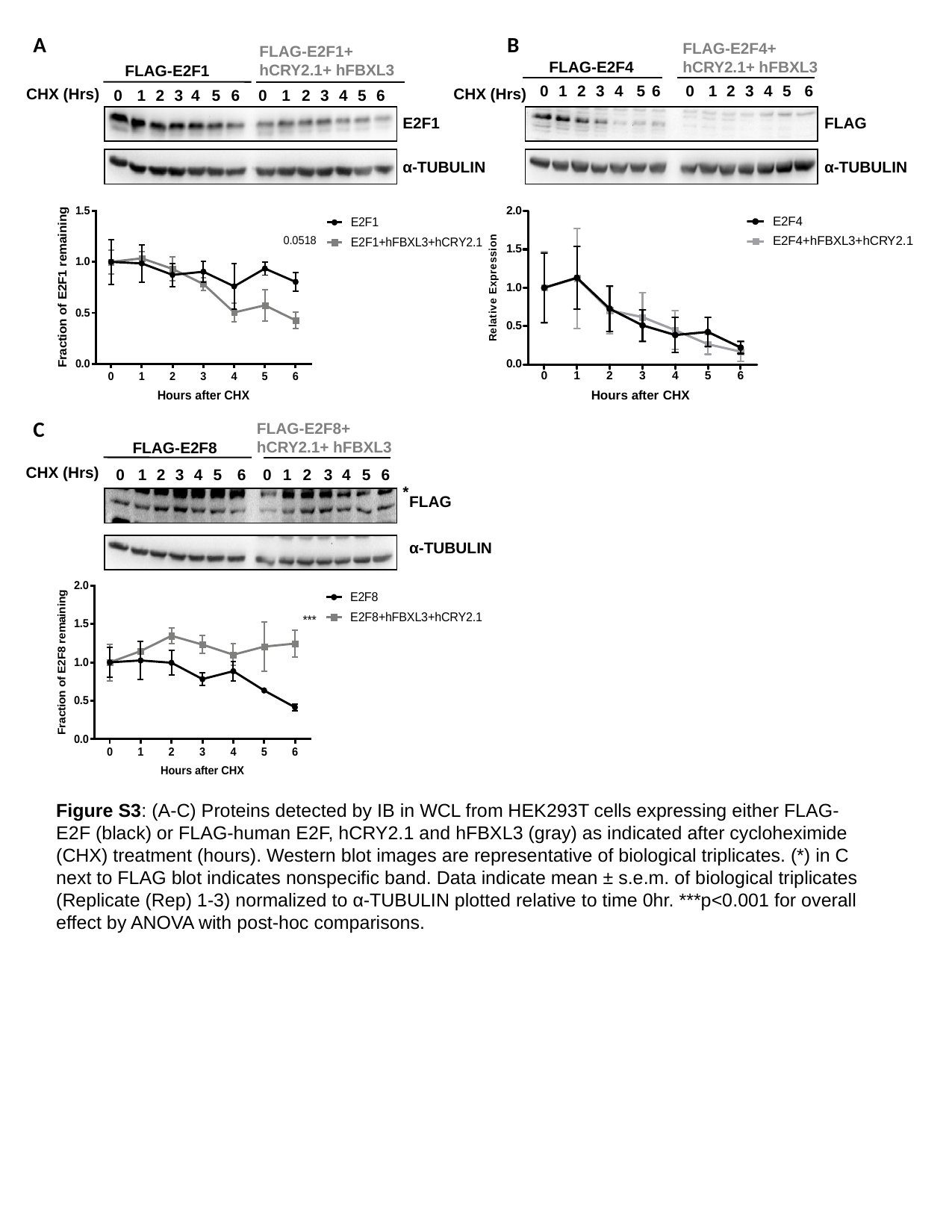

A
B
FLAG-E2F4+
hCRY2.1+ hFBXL3
FLAG-E2F1+
hCRY2.1+ hFBXL3
FLAG-E2F4
FLAG-E2F1
0
1
2
3
4
5
6
0
1
2
3
4
5
6
CHX (Hrs)
CHX (Hrs)
0
1
2
3
4
5
6
0
1
2
3
4
5
6
E2F1
FLAG
α-TUBULIN
α-TUBULIN
C
FLAG-E2F8+
hCRY2.1+ hFBXL3
FLAG-E2F8
CHX (Hrs)
0
1
2
3
4
5
6
0
1
2
3
4
5
6
*
FLAG
α-TUBULIN
Figure S3: (A-C) Proteins detected by IB in WCL from HEK293T cells expressing either FLAG-E2F (black) or FLAG-human E2F, hCRY2.1 and hFBXL3 (gray) as indicated after cycloheximide (CHX) treatment (hours). Western blot images are representative of biological triplicates. (*) in C next to FLAG blot indicates nonspecific band. Data indicate mean ± s.e.m. of biological triplicates (Replicate (Rep) 1-3) normalized to α-TUBULIN plotted relative to time 0hr. ***p<0.001 for overall effect by ANOVA with post-hoc comparisons.

## Slide 5
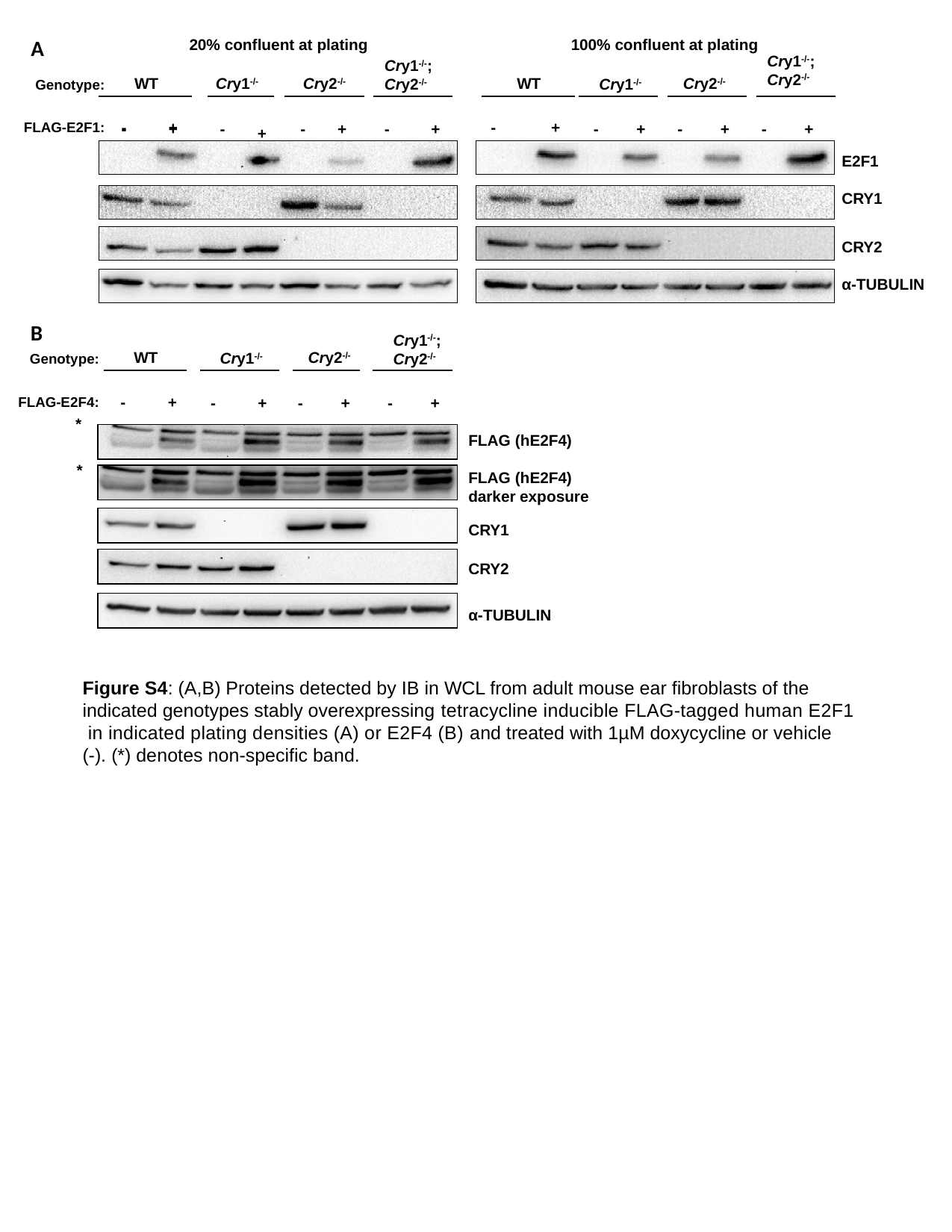

A
20% confluent at plating
100% confluent at plating
Cry1-/-; Cry2-/-
Cry1-/-; Cry2-/-
WT
Cry1-/-
WT
Cry2-/-
Cry2-/-
Cry1-/-
| Genotype: |
| --- |
| FLAG-E2F1: |
-
+
-
+
-
+
-
-
+
-
+
-
+
-
+
-
+
+
E2F1
CRY1
CRY2
α-TUBULIN
B
Cry1-/-; Cry2-/-
WT
Cry2-/-
Cry1-/-
| Genotype: |
| --- |
| FLAG-E2F4: |
-
+
-
+
-
+
-
+
*
FLAG (hE2F4)
*
FLAG (hE2F4) darker exposure
CRY1
CRY2
α-TUBULIN
Figure S4: (A,B) Proteins detected by IB in WCL from adult mouse ear fibroblasts of the indicated genotypes stably overexpressing tetracycline inducible FLAG-tagged human E2F1 in indicated plating densities (A) or E2F4 (B) and treated with 1µM doxycycline or vehicle (-). (*) denotes non-specific band.

## Slide 6
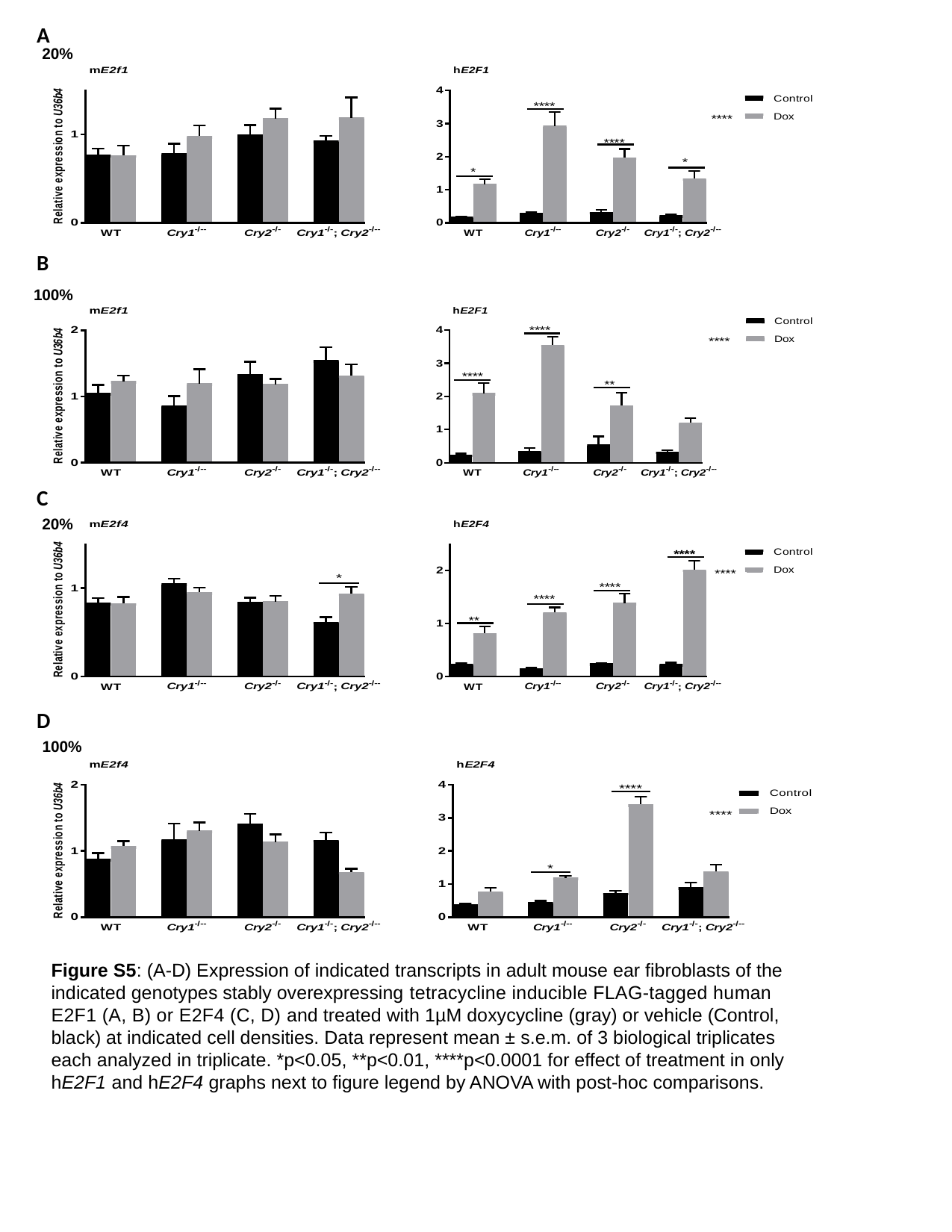

A
20%
B
100%
C
20%
D
100%
Figure S5: (A-D) Expression of indicated transcripts in adult mouse ear fibroblasts of the indicated genotypes stably overexpressing tetracycline inducible FLAG-tagged human E2F1 (A, B) or E2F4 (C, D) and treated with 1µM doxycycline (gray) or vehicle (Control, black) at indicated cell densities. Data represent mean ± s.e.m. of 3 biological triplicates each analyzed in triplicate. *p<0.05, **p<0.01, ****p<0.0001 for effect of treatment in only hE2F1 and hE2F4 graphs next to figure legend by ANOVA with post-hoc comparisons.

## Slide 7
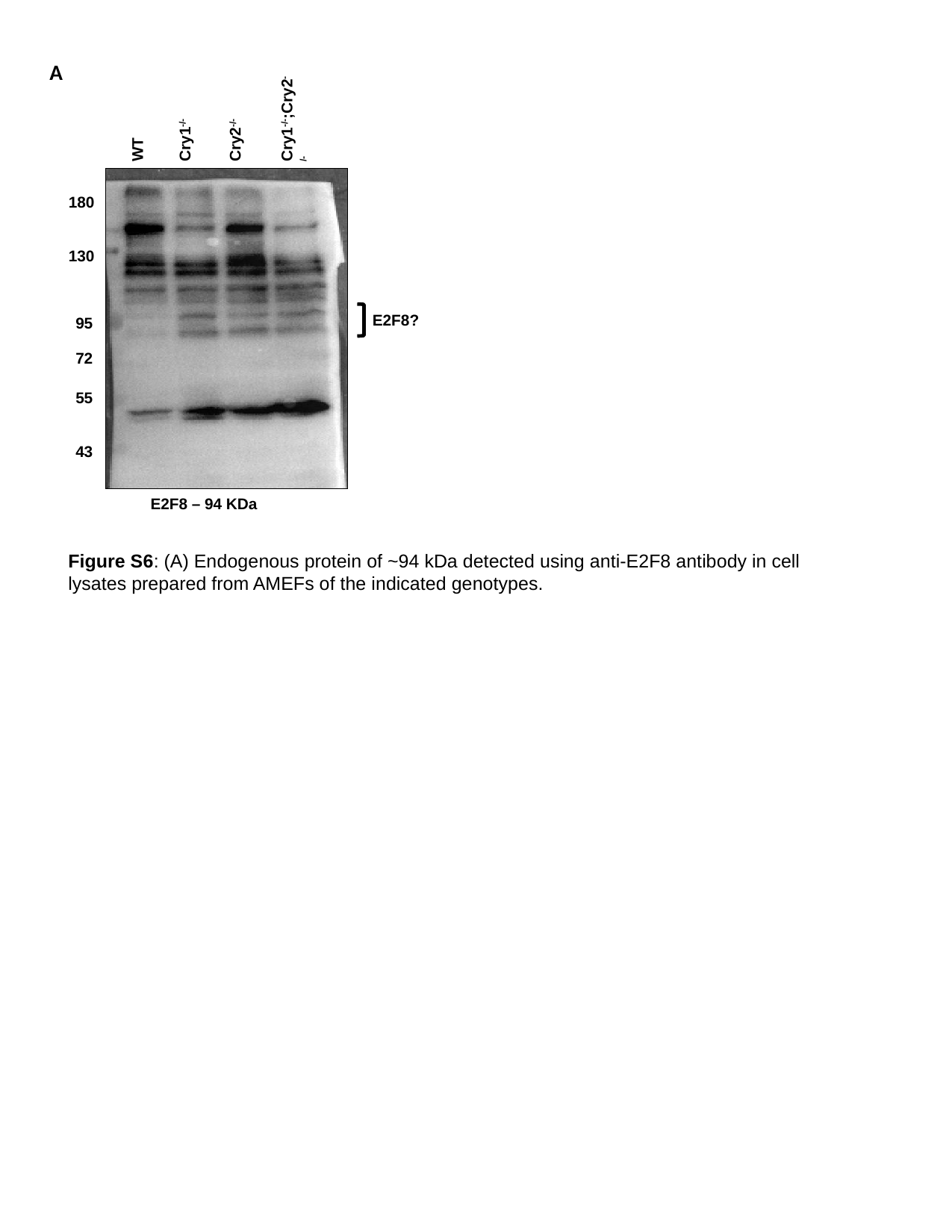

A
Cry1-/-;Cry2-/-
WT
Cry1-/-
Cry2-/-
180
130
E2F8?
95
72
55
43
E2F8 – 94 KDa
Figure S6: (A) Endogenous protein of ~94 kDa detected using anti-E2F8 antibody in cell lysates prepared from AMEFs of the indicated genotypes.

## Slide 8
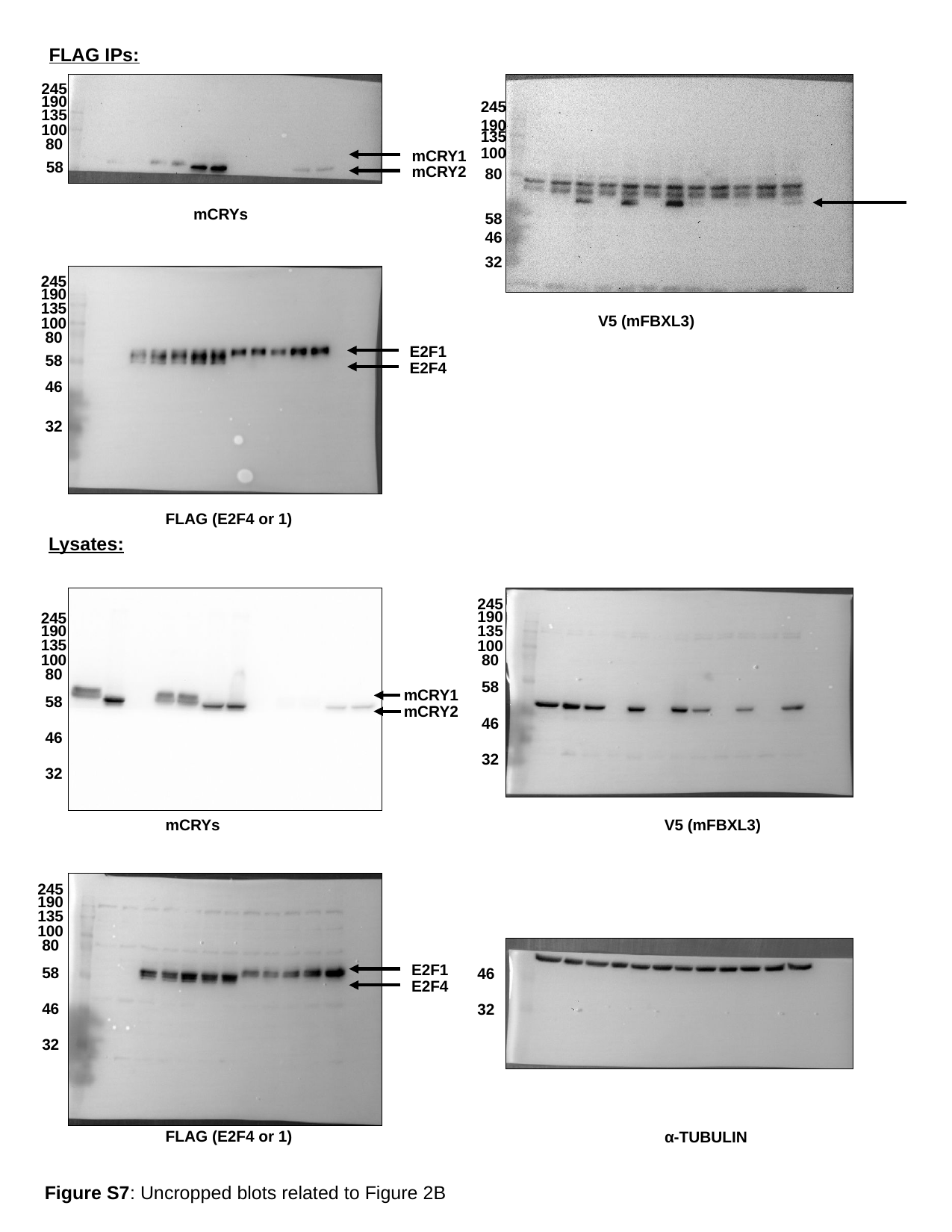

FLAG IPs:
245
190
245
135
190
100
135
80
100
mCRY1
58
mCRY2
80
mCRYs
58
46
32
245
190
135
V5 (mFBXL3)
100
80
E2F1
58
E2F4
46
32
FLAG (E2F4 or 1)
Lysates:
245
190
245
135
190
135
100
80
100
80
58
mCRY1
58
mCRY2
46
46
32
32
mCRYs
V5 (mFBXL3)
245
190
135
100
80
E2F1
58
46
E2F4
46
32
32
FLAG (E2F4 or 1)
α-TUBULIN
Figure S7: Uncropped blots related to Figure 2B

## Slide 9
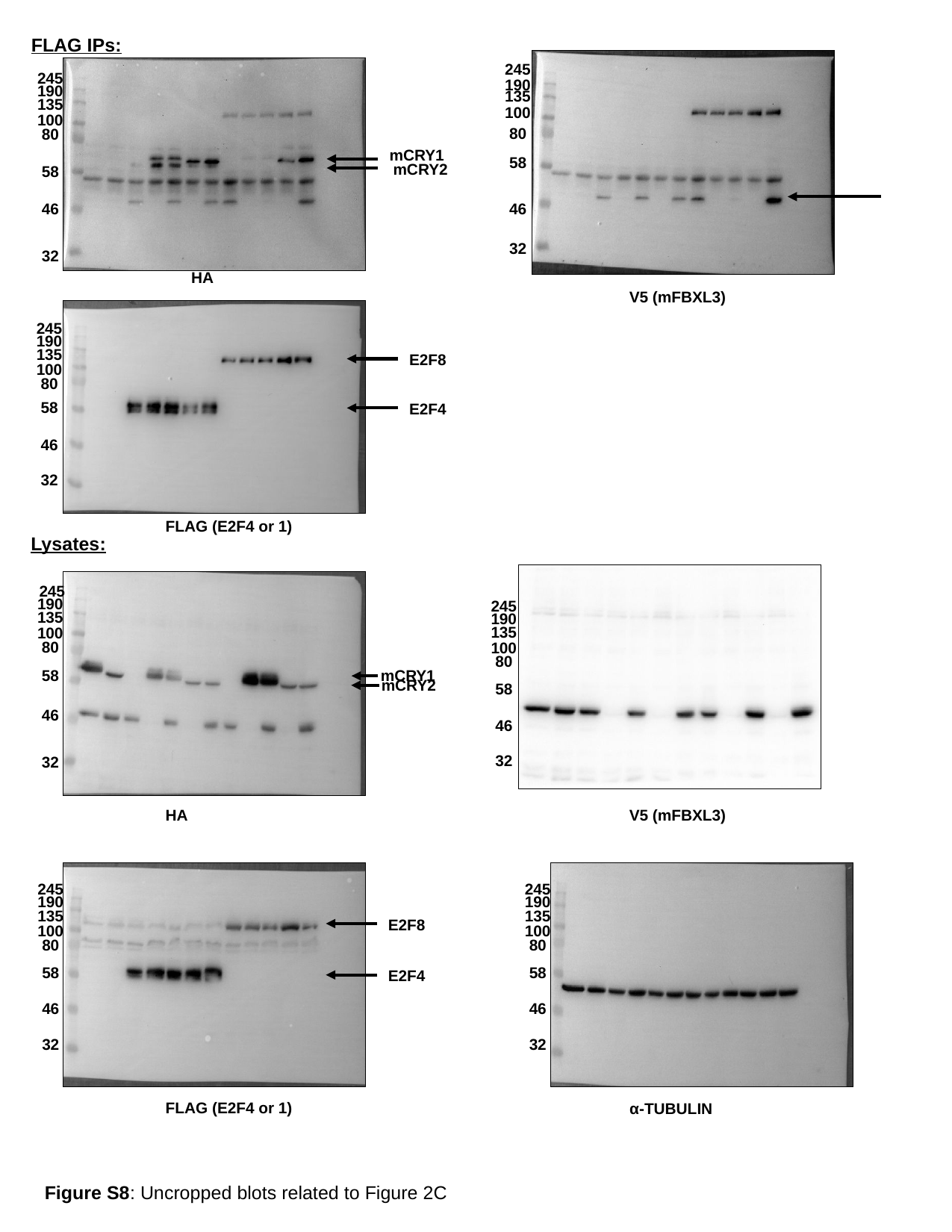

FLAG IPs:
245
245
190
190
135
135
100
100
80
80
mCRY1
58
mCRY2
58
46
46
32
32
HA
V5 (mFBXL3)
245
190
135
E2F8
100
80
58
E2F4
46
32
FLAG (E2F4 or 1)
Lysates:
245
190
245
135
190
135
100
80
100
80
58
mCRY1
mCRY2
58
46
46
32
32
HA
V5 (mFBXL3)
245
245
190
190
135
135
E2F8
100
100
80
80
58
58
E2F4
46
46
32
32
FLAG (E2F4 or 1)
α-TUBULIN
Figure S8: Uncropped blots related to Figure 2C

## Slide 10
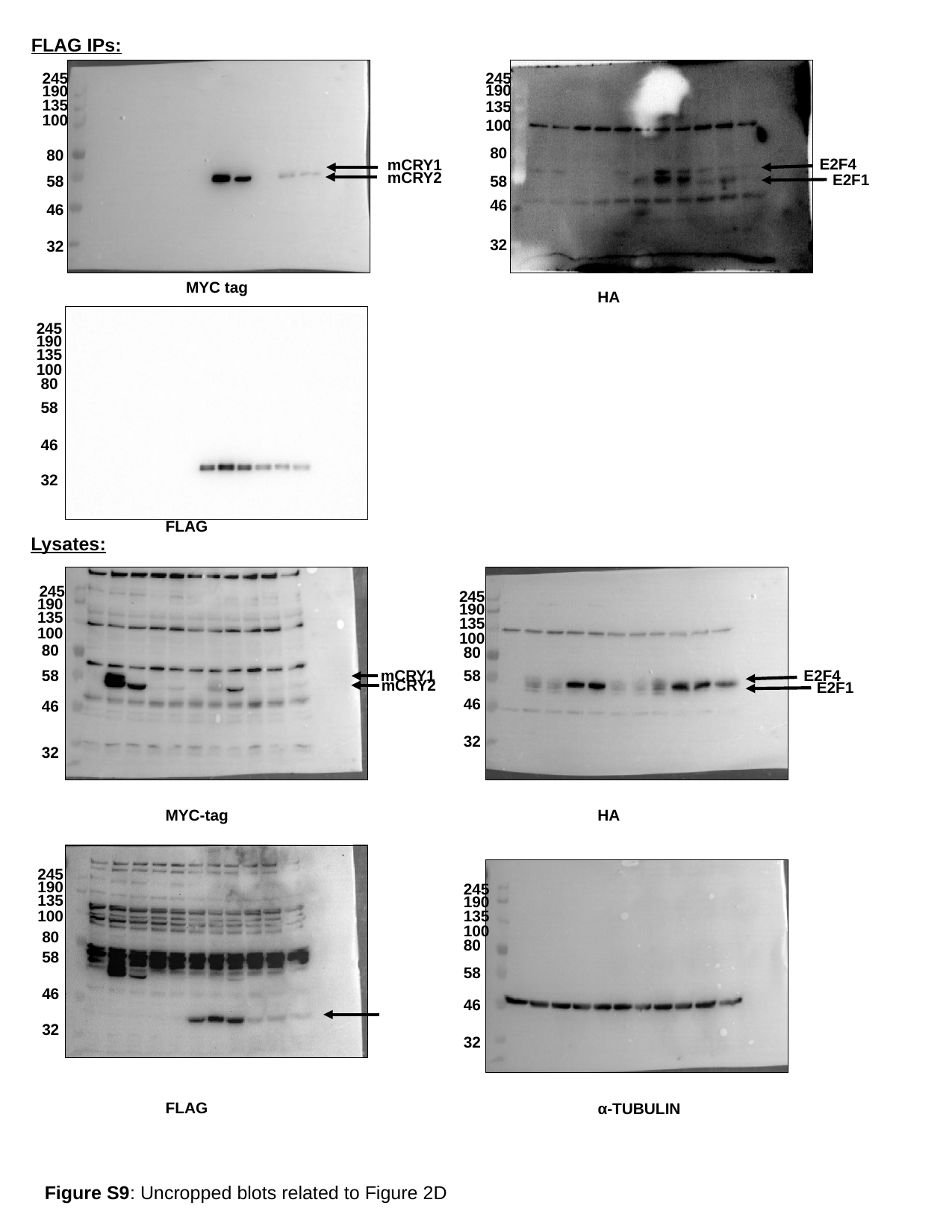

FLAG IPs:
245
245
190
190
135
135
100
100
80
80
E2F4
mCRY1
mCRY2
E2F1
58
58
46
46
32
32
MYC tag
HA
245
190
135
100
80
58
46
32
FLAG
Lysates:
245
245
190
190
135
135
100
100
80
80
58
mCRY1
58
E2F4
mCRY2
E2F1
46
46
32
32
MYC-tag
HA
245
190
245
135
190
100
135
100
80
80
58
58
46
46
32
32
FLAG
α-TUBULIN
Figure S9: Uncropped blots related to Figure 2D

## Slide 11
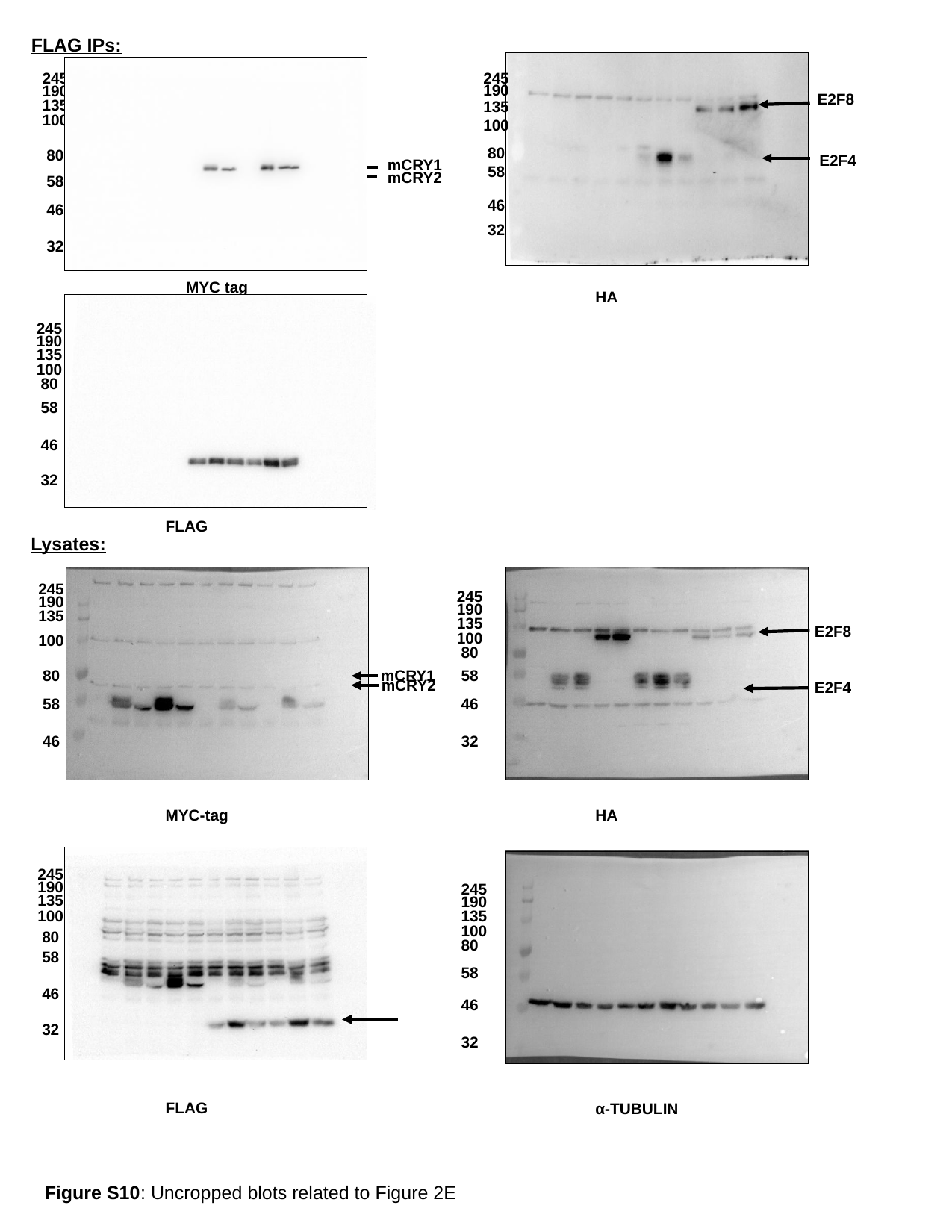

FLAG IPs:
245
245
190
190
E2F8
135
135
100
100
80
80
E2F4
mCRY1
58
mCRY2
58
46
46
32
32
MYC tag
HA
245
190
135
100
80
58
46
32
FLAG
Lysates:
245
245
190
190
135
135
E2F8
100
100
80
80
mCRY1
58
mCRY2
E2F4
58
46
46
32
MYC-tag
HA
245
190
245
135
190
100
135
100
80
80
58
58
46
46
32
32
FLAG
α-TUBULIN
Figure S10: Uncropped blots related to Figure 2E

## Slide 12
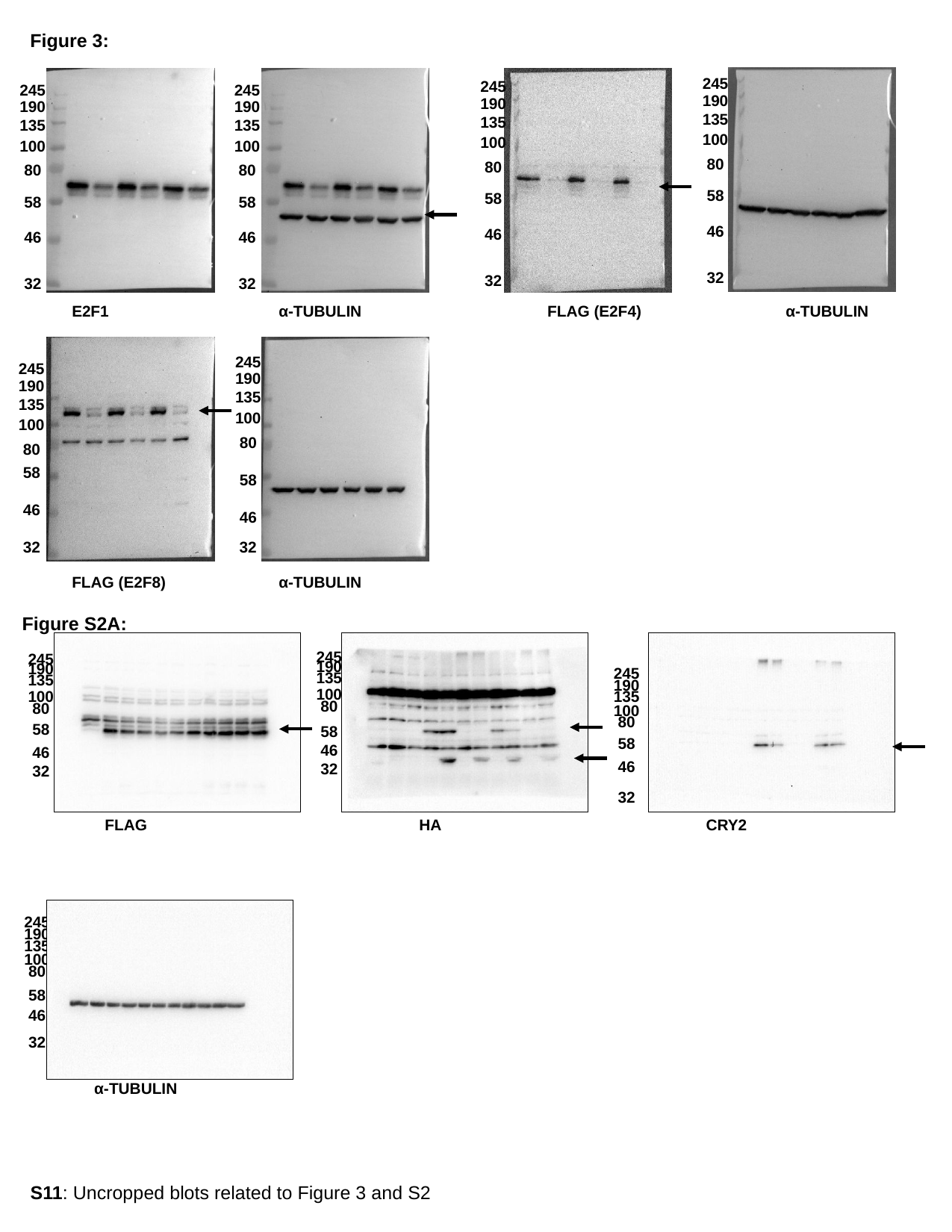

Figure 3:
245
245
245
245
190
190
190
190
135
135
135
135
100
100
100
100
80
80
80
80
58
58
58
58
46
46
46
46
32
32
32
32
E2F1
α-TUBULIN
FLAG (E2F4)
α-TUBULIN
245
245
190
190
135
135
100
100
80
80
58
58
46
46
32
32
FLAG (E2F8)
α-TUBULIN
Figure S2A:
245
245
190
190
245
135
135
190
100
100
135
80
80
100
80
58
58
58
46
46
46
32
32
32
FLAG
HA
CRY2
245
190
135
100
80
58
46
32
α-TUBULIN
S11: Uncropped blots related to Figure 3 and S2

## Slide 13
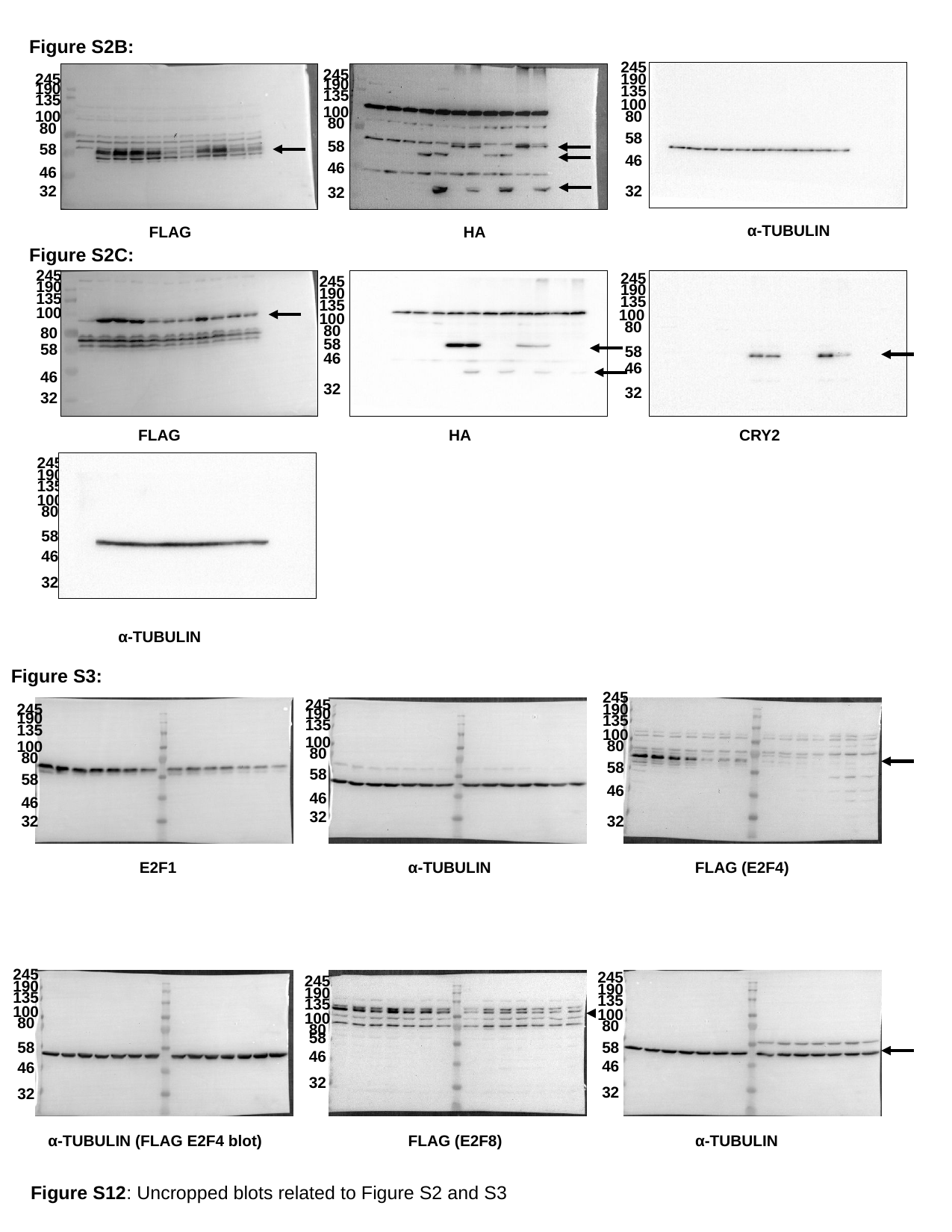

Figure S2B:
245
245
245
190
190
190
135
135
135
100
100
80
100
80
80
58
58
58
46
46
46
32
32
32
α-TUBULIN
FLAG
HA
Figure S2C:
245
245
245
190
190
190
135
135
135
100
100
100
80
80
80
58
58
58
46
46
46
32
32
32
FLAG
HA
CRY2
245
190
135
100
80
58
46
32
α-TUBULIN
Figure S3:
245
245
245
190
190
190
135
135
135
100
100
80
100
80
80
58
58
58
46
46
46
32
32
32
E2F1
α-TUBULIN
FLAG (E2F4)
245
245
245
190
190
190
135
135
135
100
100
100
80
80
80
58
58
58
46
46
46
32
32
32
α-TUBULIN (FLAG E2F4 blot)
FLAG (E2F8)
α-TUBULIN
Figure S12: Uncropped blots related to Figure S2 and S3

## Slide 14
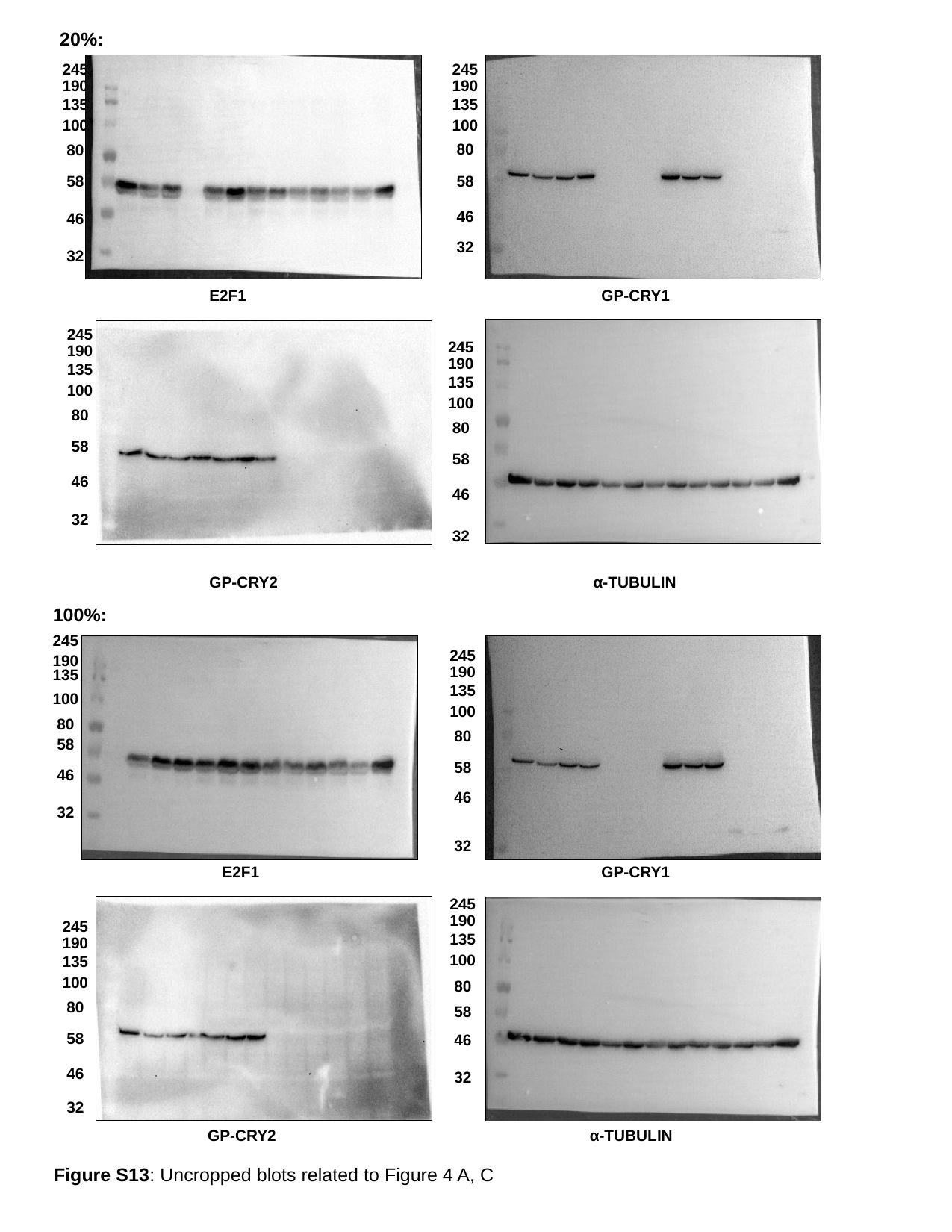

20%:
245
245
190
190
135
135
100
100
80
80
58
58
46
46
32
32
E2F1
GP-CRY1
245
190
135
100
80
58
46
32
245
190
135
100
80
58
46
32
GP-CRY2
α-TUBULIN
100%:
245
245
190
190
135
135
100
100
80
80
58
58
46
46
32
32
E2F1
GP-CRY1
245
190
245
135
190
100
135
100
80
80
58
58
46
46
32
32
GP-CRY2
α-TUBULIN
Figure S13: Uncropped blots related to Figure 4 A, C

## Slide 15
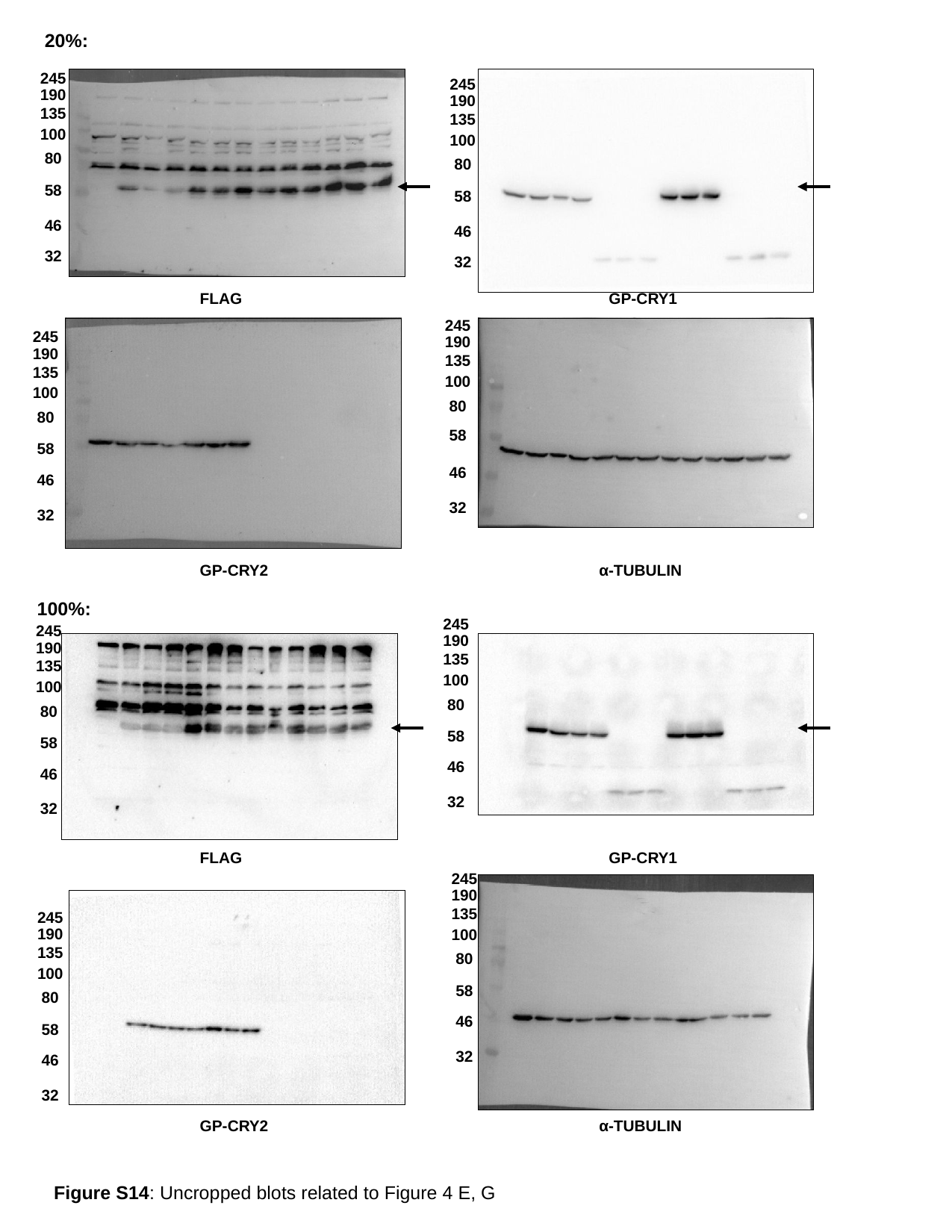

20%:
245
245
190
190
135
135
100
100
80
80
58
58
46
46
32
32
FLAG
GP-CRY1
245
245
190
190
135
135
100
100
80
80
58
58
46
46
32
32
GP-CRY2
α-TUBULIN
100%:
245
245
190
190
135
135
100
100
80
80
58
58
46
46
32
32
FLAG
GP-CRY1
245
190
245
190
135
100
80
58
46
32
135
100
80
58
46
32
GP-CRY2
α-TUBULIN
Figure S14: Uncropped blots related to Figure 4 E, G

## Slide 16
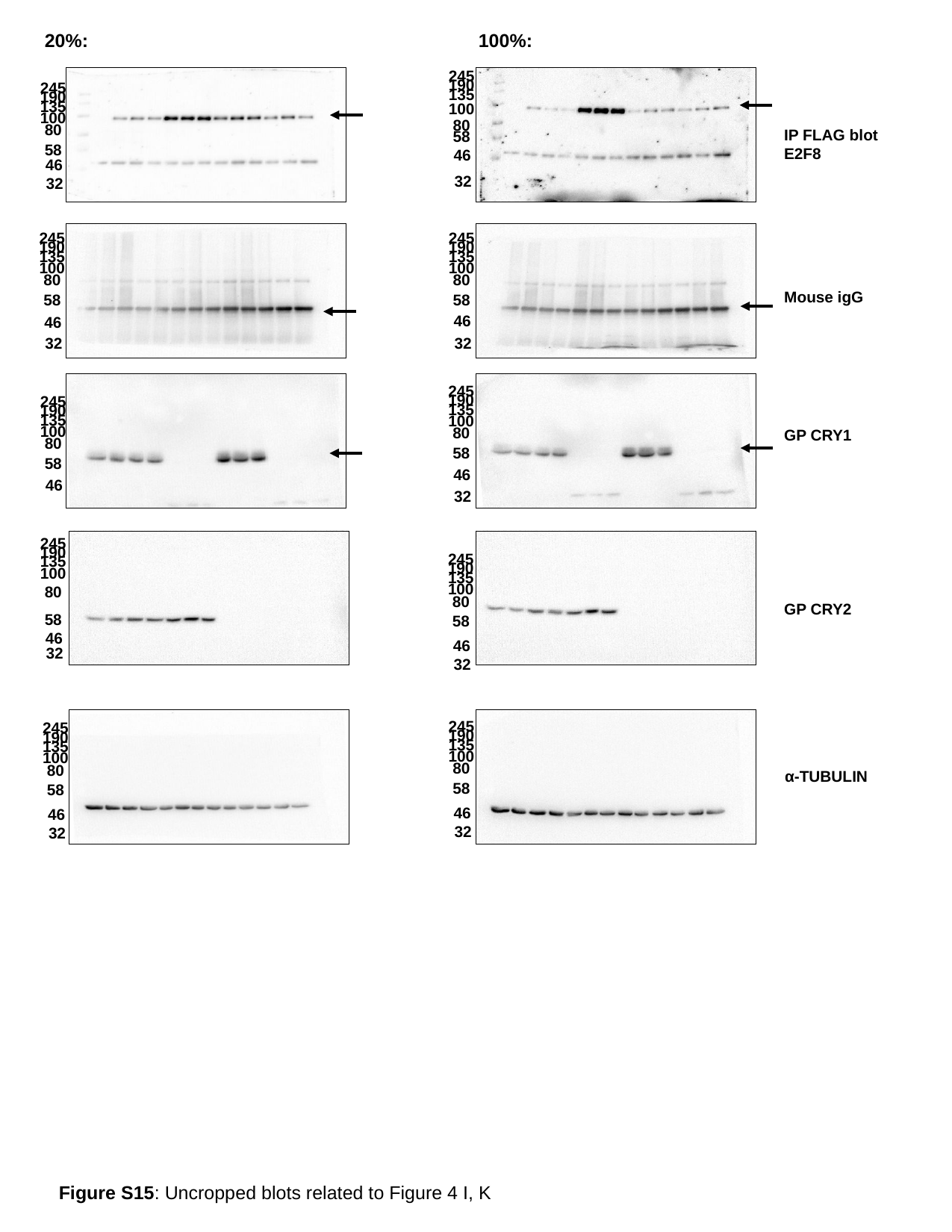

100%:
20%:
245
190
245
135
190
135
100
100
80
80
IP FLAG blot E2F8
58
58
46
46
32
32
245
245
190
190
135
135
100
100
80
80
Mouse igG
58
58
46
46
32
32
245
190
245
135
190
135
100
100
80
GP CRY1
80
58
58
46
46
32
245
190
245
135
190
100
135
100
80
80
GP CRY2
58
58
46
46
32
32
245
245
190
190
135
135
100
100
80
80
α-TUBULIN
58
58
46
46
32
32
Figure S15: Uncropped blots related to Figure 4 I, K

## Slide 17
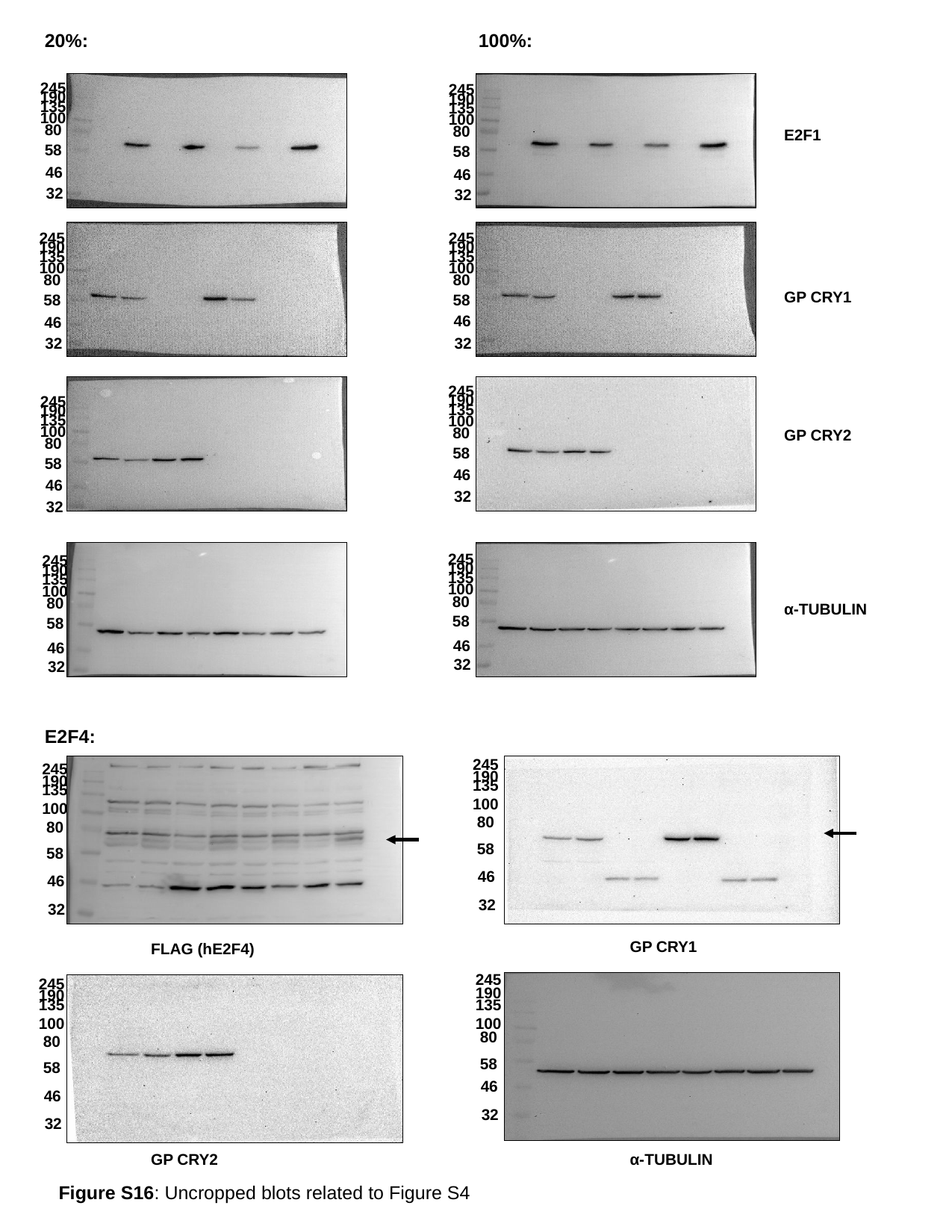

100%:
20%:
245
245
190
190
135
135
100
100
80
80
E2F1
58
58
46
46
32
32
245
245
190
190
135
135
100
100
80
80
GP CRY1
58
58
46
46
32
32
245
190
245
135
190
135
100
100
80
GP CRY2
80
58
58
46
46
32
32
245
245
190
190
135
135
100
100
80
80
α-TUBULIN
58
58
46
46
32
32
E2F4:
245
245
190
190
135
135
100
100
80
80
58
58
46
46
32
32
GP CRY1
FLAG (hE2F4)
245
245
190
190
135
135
100
100
80
80
58
58
46
46
32
32
GP CRY2
α-TUBULIN
Figure S16: Uncropped blots related to Figure S4
