# Supplementary material for: Cryptochromes modulate E2F family transcription factors
Source: Sci Rep. 2020 Mar 5;10:4077. doi: 10.1038/s41598-020-61087-y (PMC7058038; doi:10.1038/s41598-020-61087-y)
Supplement: Supplementary file 2 — Supplementary information2. [file 41598_2020_61087_MOESM2_ESM.docx]

**Table S1: E2F Hallmark Target Gene Set**

| *Lmnb1* | *Suv39h1* | *Bard1* | *Brca1* | *Msh2* | *Rec1* | *Mxd3* | *Rbbp7* |
| --- | --- | --- | --- | --- | --- | --- | --- |
| *Stmn1* | *Ezh2* | *Top2a* | *Cdc25b* | *Phe5a* | *Nup205* | *Rpa3* | *Smc6* |
| *Espl1* | *Cit* | *Asf1b* | *Rpa1* | *Rad1* | *Pola2* | *Ebe2s* | *Eif2s1* |
| *Mcm7* | *Plk4* | *Pole* | *Dek* | *Ube2t* | *Ung* | *Ranbp1* | *Mlh1* |
| *Nasp* | *Pold1* | *Kif2c* | *Dck* | *Hus1* | *E2f8* | *Prkdc* | *Pop7* |
| *Dnmt1* | *Birc5* | *Tk1* | *Cbx5* | *Cks2* | *Ppp1r b* | *Nme1* | *Pnn* |
| *Hmgb3* | *Mcm3* | *Gins1* | *Cdc20* | *Lyar* | *Myc* | *Rad51c* | *chek2* |
| *Mcm6* | *Pcna* | *Hells* | *Cenpe* | *Smc3* | *Rad50* | *Anp32e* | *Stag1* |
| *Bub1b* | *Rad51ap1* | *Lig1* | *Rfc3* | *Tubg1* | *Ctcf* | *Rocd1* | *Cdkn3* |
| *Rrm2* | *Psmc3ip* | *Mad2l1* | *Kpna2* | *Smc4* | *Ak2* | *Dclre1b* | *Prdx4* |
| *Racgap1* | *H2afz* | *Dut* | *Cdca3* | *Ccnb2* | *Pttg1* | *Asf1a* | *Brms1l* |
| *Trip13* | *Kif22* | *Cks1b* | *Ranbp1* | *Ctps* | *Nudt21* | *Ing3* | *mthfd2* |
| *Mcm2* | *Rpa2* | *Hmgb2* | *Nolc1* | *Cdk4* | *Brca2* | *Nbn* | *Nap1l1* |
| *Mcm5* | *Aurka* | *Rad21* | *Gins4* | *Nup107* | *Hn1* | *Pa2g4* | *Ipo7* |
| *Mcm4* | *Atad2* | *Ccne1* | *Psip1* | *Wdr90* | *Smc1a* | *Syncrip* | *Zw10* |
| *Ncapd2* | *Usp1* | *Mki67* | *Cenpm* | *Rnaseh2a* | *Mre11a* | *Snrpb* | *Eed* |
| *Mybl2* | *H2afx* | *Pold2* | *Ilf3* | *Pms2* | *Cdc25a* | *Nup153* | *Cdkn1a* |
| *Shmt1* | *Tacc3* | *Xrcc6* | *Gins3* | *Cse1l* | *Xpo1* | *Gspt1* | *Cdkn2a* |
| *Tmpo* | *Cdca8* | *Pold3* | *Lbr* | *Donson* | *Rec2* | *Wee1* |  |
| *Tfrc* | *Timeless* | *Tipin* | *Exosc8* | *Slbp* | *Paics* | *Hmga1* |  |
| *Chek1* | *Aurkb* | *Melk* | *Pole4* | *Tcf19* | *Tbrg4* | *Cdkn2c* |  |
| *Plk1* | *Spag5* | *Cdkn1b* | *Ssrp1* | *Hmmr* | *Prps1* | *Ppm1d* |  |

| **TABLE S2: ChIP signals from (Koike et al 2012) for E2F target genes** | | | |  |
| --- | --- | --- | --- | --- |
|  |  |  |  |  |
| Gene Name | Rank in CRY1 ChIP | MAX CRY1 ChIP signal | RANK IN CRY2 ChIP | MAX CRY2 ChIP signal |
| WEE1 | 7 | 111.69 | 377 | 12.62 |
| PRPS1 | 66 | 51.07 | 1424 | 7.78 |
| ILF3 | 96 | 42.71 | 102 | 18.93 |
| HMGA1 | 184 | 32.29 | 282 | 13.80 |
| DCTPP1 | 215 | 30.53 | 433 | 12.04 |
| CCNE1 | 297 | 26.71 | 1082 | 8.81 |
| CDK4 | 362 | 24.51 | 687 | 10.42 |
| PAICS | 427 | 22.75 | 1102 | 8.66 |
| CDC25A | 452 | 22.31 | 796 | 9.83 |
| SHMT1 | 503 | 21.13 | 958 | 9.10 |
| DDX39A | 622 | 19.08 | 2175 | 6.31 |
| CDK1 | 626 | 18.94 | 820 | 9.69 |
| NAP1L1 | 652 | 18.64 | 1979 | 6.61 |
| TK1 | 676 | 18.35 | 643 | 10.57 |
| AK2 | 977 | 15.26 | 317 | 13.36 |
| CKS2 | 1024 | 14.68 | 686 | 10.42 |
| TMPO | 1031 | 14.68 | 421 | 12.18 |
| NME1 | 1337 | 12.62 | 423 | 12.18 |
| ING3 | 1475 | 12.04 | 1146 | 8.51 |
| CDKN3 | 1488 | 12.03 | 1012 | 8.95 |
| UBE2S | 1513 | 11.89 | 310 | 13.36 |
| UNG | 1811 | 10.57 | 2379 | 6.02 |
| TUBG1 | 1859 | 10.42 | 1739 | 7.05 |
| CBX5 | 2000 | 9.98 | 3775 | 4.25 |
| DONSON | 2003 | 9.98 | 1144 | 8.51 |
| LMNB1 | 2109 | 9.54 | 2153 | 6.46 |
| PA2G4 | 2115 | 9.54 | 2484 | 5.87 |
| NOLC1 | 2125 | 9.54 | ND | 0.00 |
| TACC3 | 2127 | 9.54 | 3266 | 4.84 |
| USP1 | 2200 | 9.39 | 1067 | 8.81 |
| UBE2T | 2454 | 8.66 | 1841 | 6.90 |
| POLD2 | 2509 | 8.51 | 1303 | 8.07 |
| POLE4 | 2510 | 8.51 | 3912 | 3.96 |
| RAD50 | 2580 | 8.22 | 3053 | 5.14 |
| MXD3 | 2604 | 8.22 | ND | 0.00 |
| CCNB2 | 2673 | 8.07 | 4046 | 3.82 |
| STMN1 | 2746 | 7.93 | ND | 0.00 |
| RFC1 | 2800 | 7.78 | ND | 0.00 |
| SPC24 | 2818 | 7.78 | 933 | 9.25 |
| HNRNPD | 2885 | 7.63 | ND | 0.00 |
| CNOT9 | 2986 | 7.34 | 2680 | 5.58 |
| BIRC5 | 3073 | 7.19 | 2351 | 6.16 |
| XRCC6 | 3107 | 7.05 | 2996 | 5.14 |
| CSE1L | 3115 | 7.05 | 2228 | 6.31 |
| IPO7 | 3219 | 6.90 | ND | 0.00 |
| KPNA2 | 3221 | 6.90 | 560 | 11.15 |
| CENPM | 3368 | 6.60 | ND | 0.00 |
| BARD1 | 3384 | 6.60 | ND | 0.00 |
| ASF1B | 3453 | 6.46 | 3026 | 5.14 |
| EIF2S1 | 3472 | 6.46 | ND | 0.00 |
| AURKB | 3564 | 6.31 | 3025 | 5.14 |
| RRM2 | 3617 | 6.16 | ND | 0.00 |
| TFRC | 3649 | 6.16 | 1735 | 7.05 |
| RNASEH2A | 3709 | 6.02 | 3751 | 4.25 |
| LUC7L3 | 3762 | 6.02 | ND | 0.00 |
| DIAPH3 | 3785 | 5.87 | 4717 | 2.49 |
| ESPL1 | 3834 | 5.87 | ND | 0.00 |
| MCM7 | 3883 | 5.72 | ND | 0.00 |
| MCM5 | 3884 | 5.72 | ND | 0.00 |
| NAA38 | 3911 | 5.72 | 4572 | 2.93 |
| DLGAP5 | 3915 | 5.72 | 2500 | 5.87 |
| GINS4 | 3946 | 5.72 | ND | 0.00 |
| CKS1B | 4377 | 5.14 | 1645 | 7.34 |
| POP7 | 4394 | 4.99 | 4070 | 3.81 |
| PHF5A | 4592 | 4.70 | 468 | 11.74 |
| BRCA1 | 4852 | 4.40 | 3022 | 5.14 |
| RAN | 4917 | 4.26 | 2242 | 6.31 |
| NOP56 | 4924 | 4.26 | ND | 0.00 |
| POLD1 | 4949 | 4.26 | ND | 0.00 |
| POLA2 | 5087 | 4.11 | ND | 0.00 |
| XPO1 | 5322 | 3.81 | 4453 | 3.08 |
| HUS1 | 5349 | 3.67 | ND | 0.00 |
| NASP | 5432 | 3.67 | 4769 | 2.20 |
| MCM3 | 5602 | 3.23 | ND | 0.00 |
| STAG1 | 5637 | 3.23 | ND | 0.00 |
| TUBB | 5668 | 3.23 | ND | 0.00 |
| CDKN1B | 5705 | 3.08 | ND | 0.00 |
| GINS1 | 5723 | 3.08 | ND | 0.00 |
| KIF2C | 5746 | 2.94 | 3715 | 4.26 |
| ANP32E | 5888 | 2.64 | 4524 | 3.08 |
| NUP107 | 5906 | 2.64 | ND | 0.00 |
| NBN | 5921 | 2.50 | ND | 0.00 |
| MSH2 | 5975 | 2.35 | ND | 0.00 |
| NUP153 | 6006 | 2.20 | 3699 | 4.26 |
| CHEK2 | 6024 | 2.05 | ND | 0.00 |
| E2F8 | 6033 | 1.91 | ND | 0.00 |
| CCP110 | ND | 0.00 | 2557 | 5.87 |
| RPA2 | ND | 0.00 | 2614 | 5.72 |
| H2AFX | ND | 0.00 | 2846 | 5.43 |
| MAD2L1 | ND | 0.00 | 3073 | 5.14 |
| MYC | ND | 0.00 | 3142 | 4.99 |
| GSPT1 | ND | 0.00 | 3824 | 4.11 |
| HMGB2 | ND | 0.00 | 4089 | 3.81 |
| EXOSC8 | ND | 0.00 | 4160 | 3.67 |
| MLH1 | ND | 0.00 | 4422 | 3.23 |
| SUV39H1 | ND | 0.00 | 4618 | 2.79 |
| BUB1B | ND | 0.00 | 4681 | 2.64 |
| RFC2 | ND | 0.00 | 4743 | 2.35 |
| UBR7 | ND | 0.00 | 4821 | 1.76 |
|  |  |  |  |  |
